# Genome-wide microRNA screening reveals that the evolutionary conserved miR-9a regulates body growth by targeting sNPFR1/NPYR

**DOI:** 10.1038/ncomms8693

**Published:** 2015-07-03

**Authors:** Yoon Seok Suh, Shreelatha Bhat, Seung-Hyun Hong, Minjung Shin, Suhyoung Bahk, Kyung Sang Cho, Seung-Whan Kim, Kyu-Sun Lee, Young-Joon Kim, Walton D. Jones, Kweon Yu

**Affiliations:** 1 Neurophysiology Research Group, Bio-Nano Research Centre, Korea Research Institute of Bioscience and Biotechnology (KRIBB), 125 Gwahak-ro, Yusong-gu, Daejeon 305-806, Korea; 2 Department of Functional Genomics, Korea University of Science and Technology (UST), Daejeon 305-350, Korea; 3 Department of Biological Sciences, Korea Advanced Institute of Science and Technology (KAIST), Daejeon 305-701, Korea; 4 Department of Biological Sciences, Konkuk University, Seoul 143-701, Korea; 5 Chungnam National University Hospital, Daejeon 301-721, Korea; 6 School of Life Sciences, Gwangju Institute of Science and Technology (GIST), Gwangju 500-712, Korea

## Abstract

MicroRNAs (miRNAs) regulate many physiological processes including body growth. Insulin/IGF signalling is the primary regulator of animal body growth, but the extent to which miRNAs act in insulin-producing cells (IPCs) is unclear. Here we generate a UAS-miRNA library of *Drosophila* stocks and perform a genetic screen to identify miRNAs whose overexpression in the IPCs inhibits body growth in *Drosophila*. Through this screen, we identify *miR-9a* as an evolutionarily conserved regulator of insulin signalling and body growth. IPC-specific *miR-9a* overexpression reduces insulin signalling and body size. Of the predicted targets of *miR-9a*, we find that loss of *miR-9a* enhances the level of sNPFR1. We show via an *in vitro* binding assay that *miR-9a* binds to *sNPFR1* mRNA in insect cells and to the mammalian orthologue *NPY2R* in rat insulinoma cells. These findings indicate that the conserved *miR-9a* regulates body growth by controlling *sNPFR1/NPYR*-mediated modulation of insulin signalling.

## Abstract (web summary)

Insulin signaling governs many physiological processes but the molecular and neural mechanisms of its regulation are largely unknown. Here the authors describe a novel molecular pathway controlling sNPF regulation of insulin signalling in the fruit fly, which is mediated by the evolutionary conserved miR-9a.

## Graphical abstract

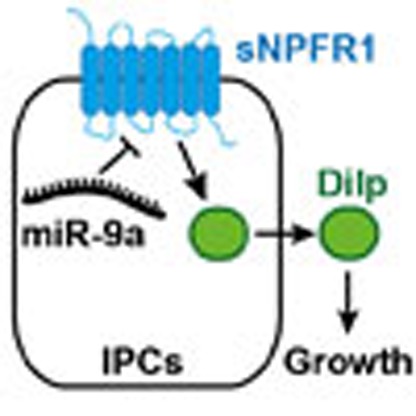

Insulin/IGF signalling is a critical pathway regulating body growth from flies to humans^1^. The *Drosophila* genome contains eight insulin-like peptides *(Dilps)* whose expression have all been confirmed via *in situ* hybridization^2^,^3^. Of the seven fly Dilps, *Dilp2, 3*, and *5* are expressed in the fly brain in the neurosecretory insulin-producing cells (IPCs). *Dilp2* seems to be the most important for proper body growth and longevity^1^,^2^ with *Dilp2* overexpression in the IPCs enhancing body growth and *Dilp2* loss-of-function inhibiting it^1^,^4^.

Neuropeptides also play important roles in the physiology and behaviours of animals. In *Drosophila*, short neuropeptide F (sNPF) regulates body growth by binding to its receptor sNPFR1 in the IPCs and activating ERK-mediated *Dilp* expression^5^,^6^. As the Dilp peptides are secreted and circulated in the body, they activate the insulin signalling cascade in target tissues resulting in body growth^2^. Thus, sNPF–sNPFR1 signalling modulates *Dilp* expression, which, in turn, modulates systemic growth, metabolism and lifespan^5^. Like the insulin signalling pathway itself, aspects of this peptidergic insulin control system are evolutionarily conserved. Mammalian neuropeptide Y receptors (NPYRs) show significant homology with sNPFR1 (ref. 7) and treatment of rat insulinoma cells with the mammalian sNPF orthologue NPY enhances their expression of insulin^5^.

MicroRNAs (miRNAs) are endogenous noncoding RNAs that act as post-transcriptional regulators of gene expression by binding to the 3′-untranslated region (UTR) of target mRNAs. miRNAs exert their effects by suppressing the translation and often inducing the degradation of their target mRNAs. miRNAs play important roles in the regulation of development, stress responses, angiogenesis, oncogenesis and diabetes^8^.

Here we report the results of a *Drosophila UAS-miRNA* library screen that identifies the conserved *miR-9a* as a regulator of insulin signalling and body growth. Furthermore, we identify *Drosophila sNPFR1* and its mammalian orthologue *NPY2R* as targets of *miR-9a* in IPCs. Since the insulin and growth phenotypes are rescued by expression of *sNPFR1,* we propose that the conserved *miR-9a/miR-9* regulates body growth by modulating insulin signalling through its conserved *sNPFR1/NPY2R* target in both *Drosophila* and mammals.

## Results

### Genome-wide miRNA screen identifies growth regulators

**Figure 1:**
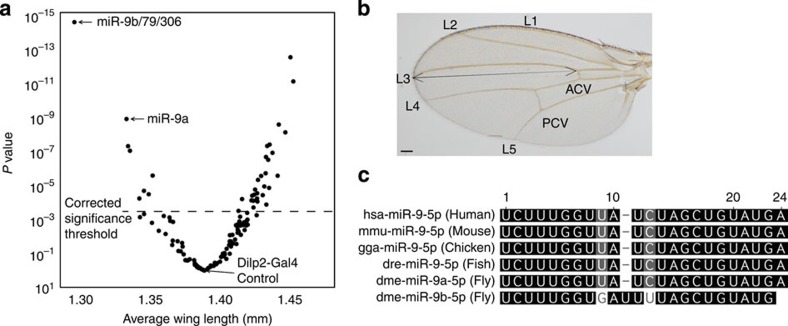
***miRNA*** **library screen identifies regulators of body growth.** (**a**) A volcano plot of the mean wing lengths of flies whose IPCs overexpress various miRNAs versus their respective *P* values derived from a one-way analysis of variance followed by pairwise *t*-tests and a Bonferroni correction for multiple comparisons. All points above the Bonferroni-adjusted significance threshold of 3.79 × 10^−4^ indicated by the dotted line can be considered significant. See Supplementary Table 2 for a full list of these data points. (**b**) Wing length was measured from the anterior cross vein (ACV) to the end of the third longitudinal vein (L3) (**c**) Mature miRNA sequences indicate the degree of conservation in the *miR-9* family in humans, mice, chickens, zebrafish and fruit flies. Scale bar, 100 μm.

To identify *miRNAs* involved in growth regulation, we generated a library of 131 *UAS-miRNA* lines covering 144 *Drosophila* miRNAs (Supplementary Fig. 1 and Supplementary Table 1 for details) and crossed them each to the brain IPC driver line *Dilp2*-*Gal4 (Dilp2>).* We then measured progeny wing length from the anterior cross vein (ACV) to the tip of the third longitudinal vein (L3) using the image analysis programme Image J (Fig. 1a,b; Supplementary Table 2). The overexpression of many *miRNAs* in the IPCs alters wing length, both positively and negatively. Figure 1a compares the mean wing length (as measured from the ACV to the tip of L3, Fig. 1b) induced by the overexpression of various *miRNAs* to their associated *P* values. The largest and most significant reductions in wing length are induced by overexpression of *miR-9a*, *miR-9b* and *miR-79*, which are all members of the conserved *miR-9* miRNA family (Fig. 1c). The larger reduction in wing length observed for the *UAS-miR-9b/79/306* line, which induces overexpression of both *miR-9b* and *miR-79*, is consistent with the fact that both *miR-9b* and *miR-79* have very similar sequences and are likely capable of regulating the same targets. Since *UAS-miR-9a* significantly reduces wing length via overexpression of a single miRNA, simplifying subsequent analyses, we focused the rest of our efforts on *miR-9a*.

### *miR-9a* is expressed in the insulin-producing cells of brains

**Figure 2:**
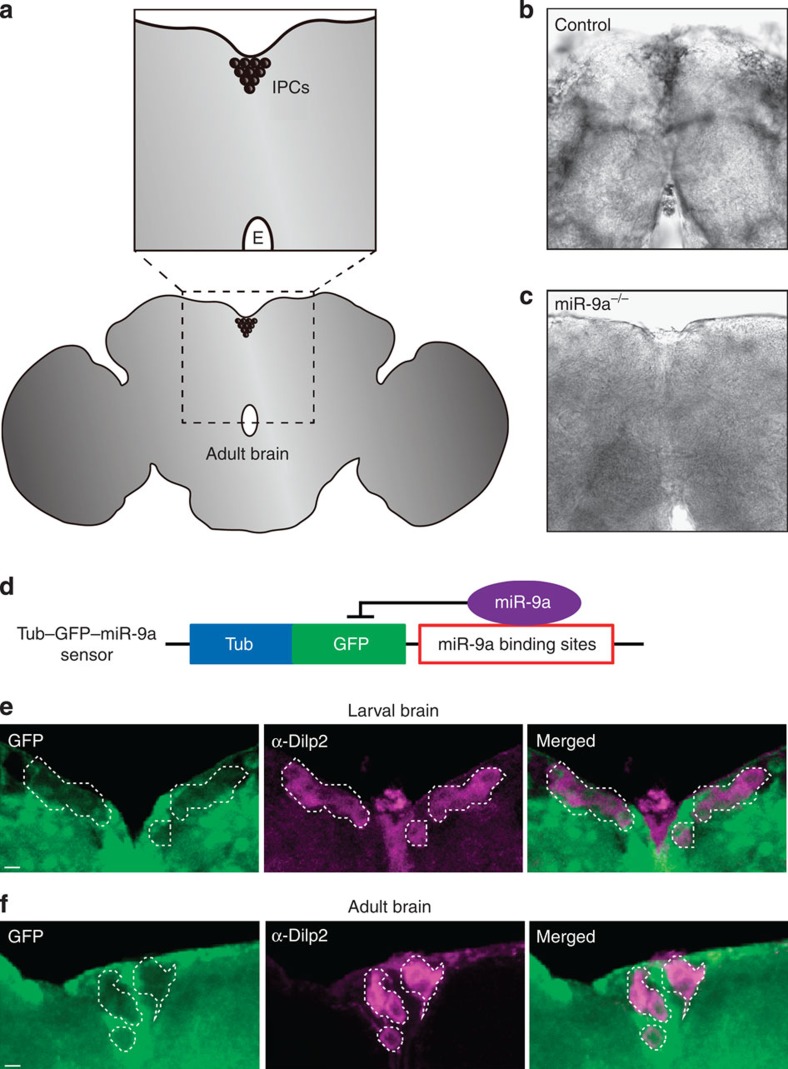
***miR-9a*** **is expressed in the insulin-producing cells.** (**a**) Adult brain schematics indicate the position of the IPCs in the median neurosecretory cluster in the *pars intercerebralis*. The ‘E' indicates the oesophagus. An *in situ* hybridization with an LNA probe specific to the mature *miR-9a* sequence stains the IPCs in the *w*^*1118*^ control genotype (**b**), but not in *miR-9a*^*E39/J22*^ *null mutant* (*miR-9a*^*−/−*^) brains (**c**). (**d**) A *Tubulin–GFP–miR-9a* sensor schematic. (**e**,**f**) The *Tubulin–GFP–miR-9a* sensor shows reduced GFP fluorescence in both the larval (**e**) and adult (**f**) IPCs, which are marked by an anti-Dilp2 antibody and with dotted white lines. Scale bar, 10 μm.

To investigate the mechanism by which *miR-9a* alters growth, we used a locked nucleic acid (LNA) probe specific to the mature form of *miR-9a* in an *in situ* hybridization experiment. This allowed us to visualize *miR-9a* expression in larval and adult brains. The IPCs comprise a bundle of bilaterally symmetric neurosecretory cells located dorsally along the midline of the protocerebrum in a region called the pars intercerebralis (PI) (Fig. 2a). We observed staining associated with *miR-9a* probe binding in both the larval (Supplementary Fig. 2) and adult PI regions in a control genotype (*w*^*1118*^) (Fig. 2b; Supplementary Fig. 2b), but not in homozygous *miR-9a*^*−/−*^ null mutant brains stained under identical conditions (Fig. 2c; Supplementary Fig. 2c). Although this staining seems to be widespread, its absence in the *miR-9a* mutants is consistent with some level of endogenous *miR-9a* expression in the IPCs of both larvae and adults (Fig. 2; Supplementary Fig. 2). To further verify the expression of *miR-9a* in larval and adult IPCs, we examined *Tubulin–GFP–miR-9a* sensor lines^9^ (Fig. 2d) whose ubiquitous expression of GFP is suppressed in *miR-9a*-expressing cells. The larval and adult IPCs in the *Tubulin–GFP–miR-9a* sensor flies, here identified by a Dilp2-specific antibody, exhibit less GFP staining than surrounding cells (Fig. 2e,f). Thus, both our *in situ* hybridization and *Tubulin–GFP–miR-9a* sensor results demonstrate *miR-9a* expression in both larval and adult IPCs.

### *miR-9a* regulates growth and insulin signalling

**Figure 3:**
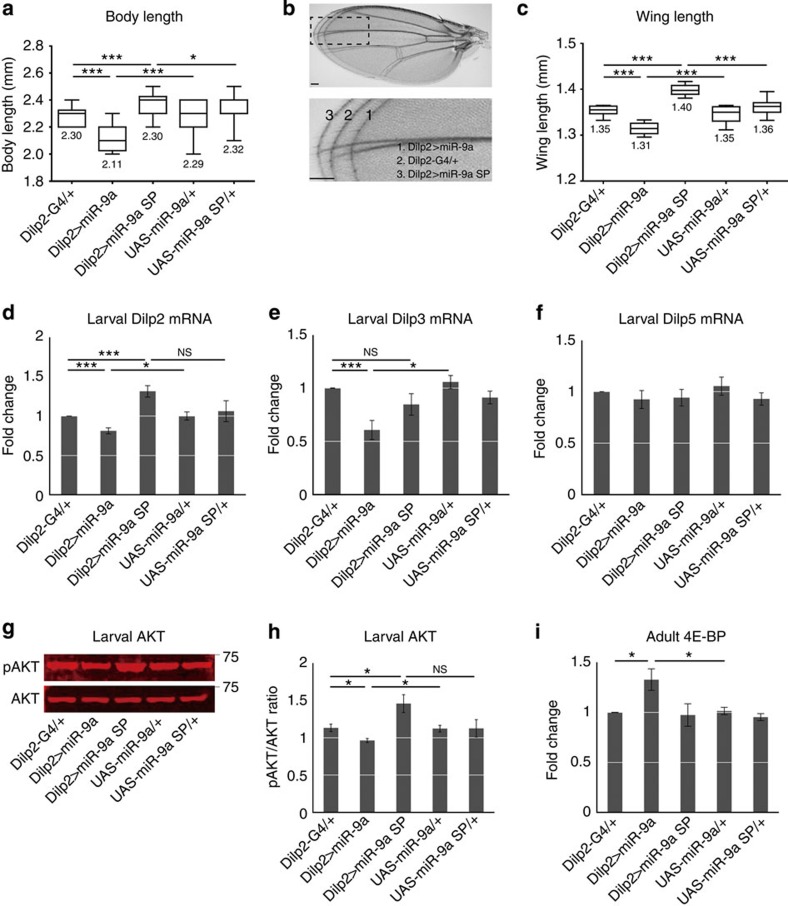
***miR-9a*** **regulates growth and insulin signalling.** (**a**) When compared with the heterozygous *Dilp2-Gal4/+*, *UAS-miR-9a/+* and *UAS-miR-9a sponge/+* controls, overexpression of *miR-9a* in the IPCs (*Dilp2>miR-9a*) reduces body length, while *miR-9a* knockdown via a *miR-9a sponge* (*Dilp2>miR-9a sponge*) increases body length. (**b**) Superimposed wing images demonstrate the degree to which *miR-9a* modulates wing size. (**b**,**c**) Overexpression of *miR-9a* reduces wing length, while *miR-9a* knockdown increases wing length. (**d**–**f**) IPC-specific *miR-9a* overexpression reduces larval expression of *Dilp2 and 3* (*Dilp2>miR-9a*), while *miR-9a* knockdown increases larval expression of *Dilp2* (*Dilp2>miR-9a sponge*). Values are normalized to the *Dilp2-Gal4/+* control. *Dilp5* levels remain unchanged by *miR-9a* manipulation. (**g**,**h**) Fluorescent western blot visualization of pAKT versus AKT. Larval IPC-specific *miR-9a* overexpression (*Dilp2>miR-9a*) reduces activated pAKT, while *miR-9a knockdown* (*Dilp2>miR-9a sponge*) increases activated pAKT. (**i**) Adult IPC-specific overexpression of *miR-9a* increases *4E-BP* levels compared with the *Dilp2-Gal4/+* and *UAS-miR-9a/+* controls. Data in **d**–**i** are presented as mean±s.e.m. from at least three independent experiments. Statistical significance was assessed by two-tailed Student's *t*-test; NS, not significant, *P<0.05, ***P<0.001. Scale bar, 100 μm.

We next increased *miR-9a* expression in the IPCs using *UAS-miR-9a* (*Dilp2>miR-9a*) and reduced *miR-9a* expression in the IPCs using *UAS-miR-9a sponge* (*Dilp2>miR-9a sponge*). The *UAS-miR-9a* *sponge* contains 10 × *miR-9a* binding sites and tissue specifically reduces *miR-9a* levels by soaking up endogenous *miR-9a* molecules^10^. We found *miR-9a* overexpression in the IPCs (*Dilp2>miR-*9a) reduces body length, wing length and pupal volume, while *miR-9a* knockdown in the IPCs (*Dilp2>miR-9a sponge*) increases body length, wing length and pupal volume (Fig. 3a–c; Supplementary Fig. 3a). Since insulin/IGF signalling is a major determinant of body growth from flies to human^1^, we tested *Dilp* levels in these same flies. Although manipulation of *miR-9a* does not alter larval *Dilp5* expression, IPC overexpression of *miR-9a* (*Dilp2>miR-9a*) reduces larval *Dilp2* and *Dilp3* mRNA levels, while reduction of *miR-9a* (*Dilp2>miR-9a sponge*) significantly increases larval *Dilp2* mRNA levels (Fig. 3d–f). Consistently, IPC overexpression of *miR-9a* (*Dilp2>miR-9a*) also reduces *Dilp2, 3 and 5* mRNA levels, while reduction of *miR-9a* (*Dilp2>miR-9a sponge*) significantly increases *Dilp2* mRNA levels (Supplementary Fig. 4a,c,e) in adult heads. Thus, *miR-9a* in the IPCs seems to regulate body growth via modulation of insulin expression.

We next measured AKT phosphorylation and the expression of the dFOXO target gene *4E-BP* in an effort to assess insulin signalling in insulin target tissues. When insulin signalling is activated in the target tissues, AKT phosphorylation is increased and *4E-BP* expression is reduced because of the reduced nuclear localization of dFOXO. As expected, overexpression of *miR-9a* in the IPCs (*Dilp2>miR-9a*) reduces the level of pAKT in both larvae (Fig. 3g,h) and adults (Supplementary Fig. 5a) and increases the level of *4E-BP* in adults (Fig. 3i). Reduction of *miR-9a* in the IPCs *(Dilp2>miR-9a sponge*) increases the level of pAKT in larvae (Fig. 3g,h). In addition, the levels of haemolymph glucose and trehalose rise with *miR-9a* overexpression and fall with *miR-9a* knockdown (Supplementary Fig. 6a). These results indicate that *miR-9a* modulates body growth and metabolism by regulating IPC insulin expression and consequently insulin signalling in peripheral target tissues.

**Figure 4:**
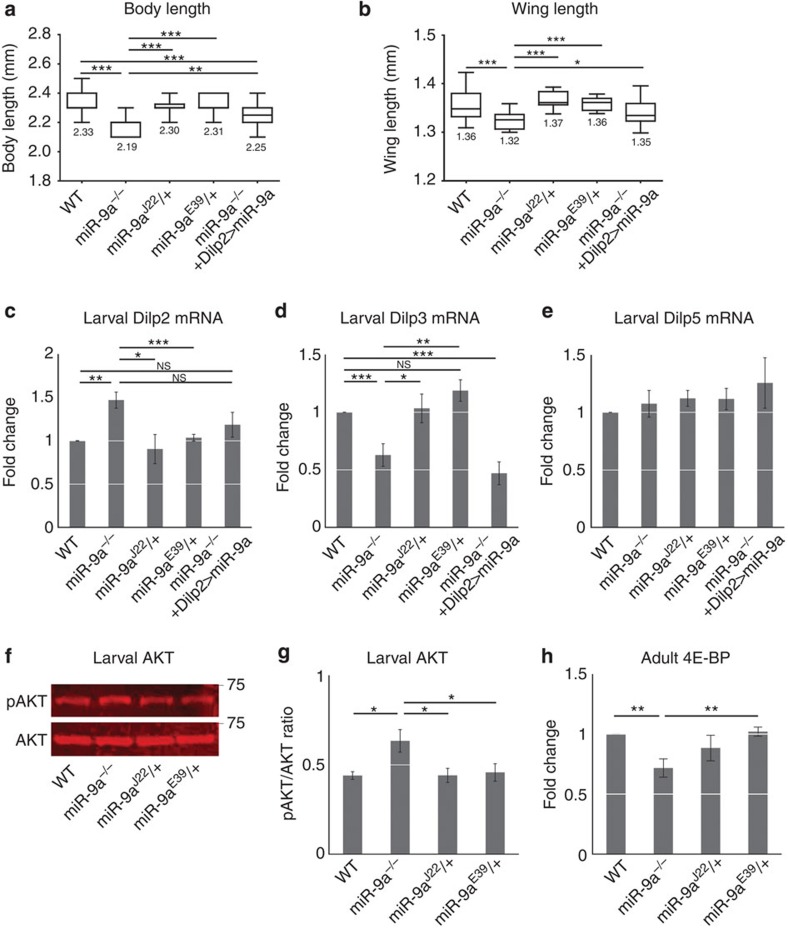
IPC-specific *miR-9a*overexpression rescues *Dlip2* expression of the *miR-9a* null mutant. (**a**,**b**) Transheterozygous *miR-9a*^*E39/J22*^ null mutants show reduced body and wing size compared with *WT, miR-9a*^*J22*^*/+* and *miR-9a*^*E39*^*/+* heterozygous controls, but this growth defect is rescued via IPC-specific *miR-9a* overexpression (*miR9a*^*−/−*^*+Dilp2>miR-9a*). (**c**–**e**) *miR-9a*^*E39/J22*^ null mutants have higher larval *Dilp2* expression and lower *Dilp3* expression than *WT, miR-9a*^*J22*^*/+* and *miR-9a*^*E39*^*/+* heterozygous controls. *Dilp2* expression is restored in *miR-9a*^*E39/J22*^ null mutants by simultaneous IPC-specific *miR-9a* overexpression (*miR9a*^*−/−*^*+Dilp2>miR-9a*), but *Dilp3* expression is further reduced. (**f**,**g**) Larval activated pAKT is elevated in *miR-9a*^*E39/J22*^ null mutants when compared with *WT, miR-9a*^*J22*^*/+* and *miR-9a*^*E39*^*/+* heterozygous controls. (**h**) Adult *4E-BP* expression is reduced in the *miR-9a*^*E39/J22*^ null mutants compared with *WT, miR-9a*^*J22*^*/+* and *miR-9a*^*E39*^*/+* heterozygous controls. Data are presented as mean±s.e.m. from at least three independent experiments. Statistical significance was assessed by two-tailed Student's *t*-test; NS, not significant, *P<0.05, **P<0.01, ***P<0.001.

In further confirmation of our findings, *Dilp2* expression in *miR-9a*^*−/−*^ null mutant flies is increased like in flies whose IPCs express the *miR-9a sponge* (Fig. 4c). Similar to our findings in *Dilp2>miR-9a sponge* flies, pAKT levels are increased and *4E-BP* levels are decreased in the insulin target tissues of *miR-9a*^*−/−*^ null mutants (Fig. 4f–h; Supplementary Fig. 5b). Glucose and trehalose are also reduced in *miR-9a*^*−/−*^ null mutant haemolymph as in the *Dilp2>miR-9a sponge* flies (Supplementary Fig. 6b). The body length, wing length and pupal volume of *miR-9a*^*−/−*^ null mutants, however, are all reduced (Fig. 4a,b; Supplementary Fig. 3b), in seeming contrast with the results we observed in the *Dilp2>miR-9a sponge* flies. This suggests that *miR-9a* in non-IPCs may affect body growth differently than *miR-9a* in the IPCs. In addition, we show that IPC-specific *miR-9a* overexpression rescues the *Dilp2* expression of the null mutant (Fig. 4c–e; Supplementary Fig. 4b,d,f).

**Figure 5:**
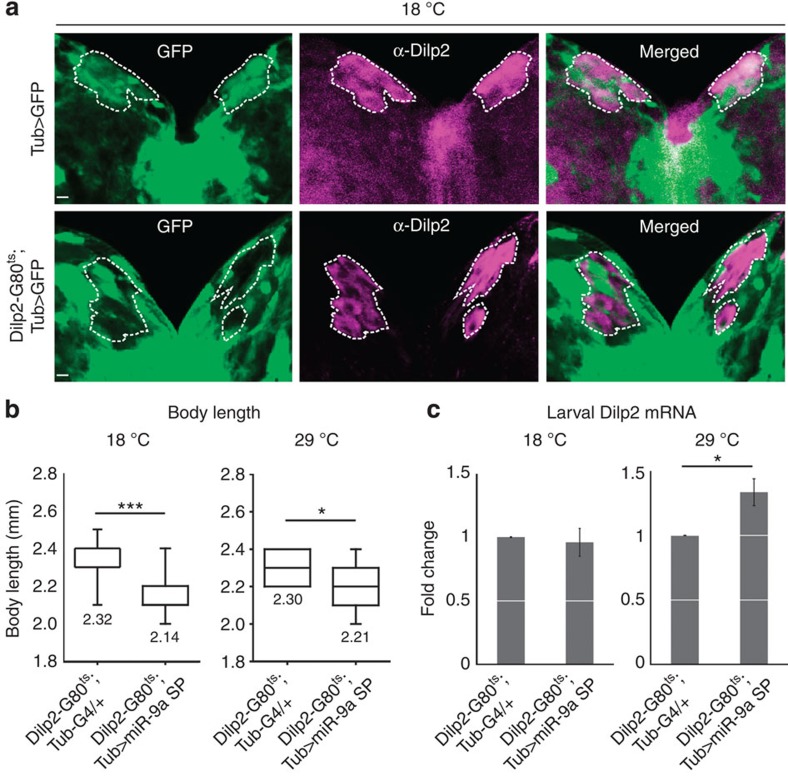
***miR-9a*** **inside and outside the IPCs** **differentially affects body growth.** (**a**) At the permissive temperature of 18 °C, the *Dilp2-Gal80*^*ts*^ transgene blocks ubiquitous GFP expression (*Tub>GFP)* in the larval IPCs, as indicated by anti-Dilp2 staining (magenta). (**b**,**c**) Suppression of *miR-9a* expression outside the IPCs (*Dilp2-G80*^*ts*^*; Tub>miR-9a sponge*, 18 °C) significantly reduces body length but does not affect *Dilp2* expression. When *Dilp2-Gal80*^*ts*^ is inactivated at 29 °C and *miR-9a* is ubiquitously suppressed (*Dilp2-G80*^*ts*^*; Tub>miR-9a sponge*), body length is less significantly reduced but *Dilp2* expression is increased. Data are presented as mean±s.e.m. from at least three independent experiments. Statistical significance was assessed by two-tailed Student's *t*-test, *P<0.05, ***P<0.001. Scale bar, 10 μm.

To further support the differential effects of *miR-9a* in the IPCs and in non-IPCs on body growth, we generated a *Dilp2-Gal80*^*ts*^ line that suppresses Gal4 activity in the IPCs at the 18 °C permissive temperature but not at the 29 °C restrictive temperature. We first verified the function of the *Dilp2-Gal80*^*ts*^ line by confirming that it specifically suppresses in the larval IPCs, the otherwise ubiquitous GFP signal conferred by *Tub>GFP* (Fig. 5a). When *miR-9a* expression is suppressed outside the IPCs (*Dilp2-G80*^*ts*^*; Tub>miR-9a sponge*, 18 °C), body length is significantly reduced compared with the *Dilp2-G80*^*ts*^*; Tub-Gal4* control but *Dilp2* expression is unchanged (Fig. 5b,c). When *miR-9a* is suppressed everywhere by shifting the *Dilp2-G80*^*ts*^*; Tub>miR-9a sponge* flies to the restrictive temperature, body length is also reduced and *Dilp2* expression is increased like the phenotype we observed in *miR-9a*^*−/−*^ null mutant flies (compare Fig. 5b,c with Fig. 4a,c). These data reveal that *miR-9a* in the IPCs and in non-IPCs affect body growth differently.

### *sNPFR1* and its orthologue *NPY2R* are targets of *miR-9a*

Since the sequence of mature *miR-9a* is well-conserved from flies to humans (Fig. 1c), we reasoned that the *miR-9a* target responsible for the growth control phenotype may also be conserved. Of the candidate *miR-9a* targets predicted by Targetscan (www.targetscan.org) and miRanda (www.microRNA.org), we selected *sNPFR1* and its mammalian orthologue *NPY2R* for further analysis because of their conservation and known regulation of body growth via modulation of insulin expression^5^,^7^.

**Figure 6:**
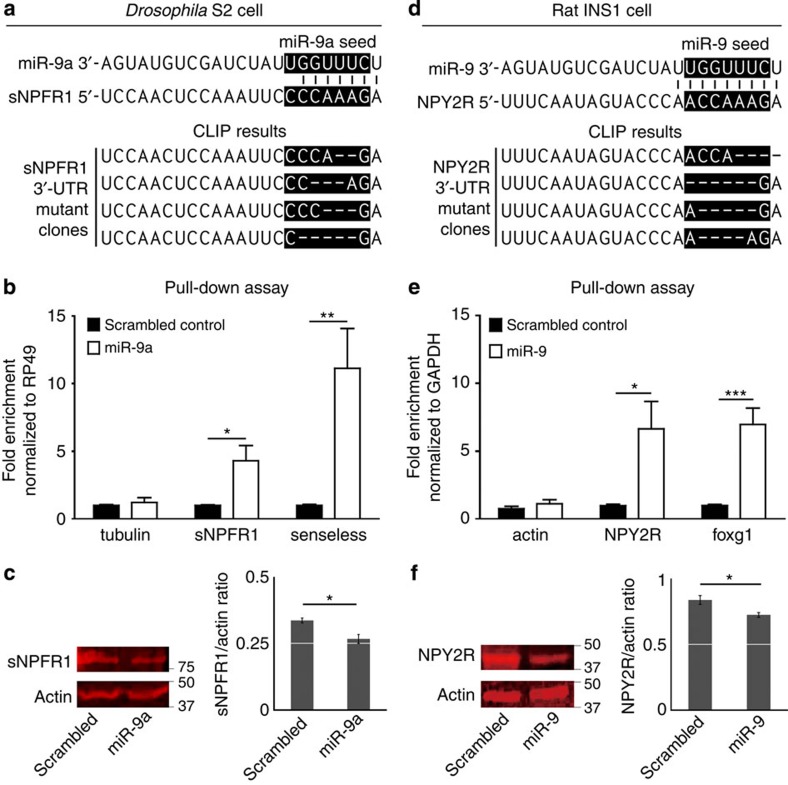
***miR-9a*** **regulates the insulin modulator** ***sNPFR1*** **and its mammalian orthologue** ***NPY2R***. (**a**,**d**; above) Alignment of the mature *miR-9a* sequence with its predicted target sequences in the 3′-UTRs of *sNPFR1* and *NPY2R.* (**a**,**d**; below) Mutant *sNPFR1* and *NPY2R* 3′-UTR clones induced in CLIP assays in *Drosophila* S2 and rat INS-1 cells, respectively. (**b**,**e**) miRNA–mRNA–RISC pull-down assays in *Drosophila* S2 (**b**) and rat INS-1 (**e**) cells reveal clear binding of *miR-9a* to both the *sNPFR1* and *NPY2R* mRNAs. Transfection of *miR-9a* leads to enrichment of *sNPFR1* and *NPY2R* mRNAs just like known target mRNAs *senseless* and *foxg1* when normalized to *rp49* and *GAPDH* levels. Transfection with a scrambled version of *miR-9a* (Supplementary Table 3) causes no such enrichment. (**c**,**f**) Transfection of a synthetic *miR-9a* duplex reduces the level of sNPFR1 protein produced by S2 cells (**c**) and the level of NPY2R protein produced by INS-1 cells (**f**) when compared with levels produced on transfection with a scrambled *miR-9a* (Supplementary Table 3). Data in **b**,**c** and **e**,**f** are presented as mean±s.e.m. from at least three independent experiments. Statistical significance was assessed by two-tailed Student's *t*-test, *P<0.05, **P<0.01, ***P<0.001.

The 3′-UTRs of both *sNPFR1* and *NPY2R* have predicted *miR-9a* binding sites (Fig. 6a,d). We performed a cross-linking immunoprecipitation (CLIP) assay to determine whether *miR-9a* binds directly to the *sNPFR1* and *NPY2R* 3′-UTRs. In the CLIP technique, miRNA-directed Argonaute-1 (AGO1) binding to a target mRNA followed by ultraviolet cross-linking, immuno-precipitation and subsequent proteinase treatment results in molecules of mRNA cross-linked to short AGO1-derived peptides that interfere with proper reverse transcription to complementary DNA (cDNA). This means 3′-UTR fragment clones of legitimate *miRNA* targets contain small deletions surrounding the site of AGO1 cross-linking, which is often in or near the *miRNA* seed sequence crucial for target mRNA pairing^11^,^12^. We performed the CLIP assay on *Drosophila* S2 and rat INS-1 cells transfected with *miR-9a* or a scrambled synthetic *miRNA*. We were able to observe several two to six nucleotide deletions in the *miR-9a* seed sequence matches of random *sNPFR1* and *NPY2R* 3′-UTR clones from both cell types transfected with *miR-9a* (Fig. 6a,d; Supplementary Table 5). This confirms that *miR-9a* binds to the 3′-UTRs of *sNPFR1* and *NPY2R*.

Next, we performed a miRNA–mRNA pull-down assay for further confirmation of *miR-9a* binding to the *sNPFR1* and *NPY2R mRNAs.* After transfection with either biotinylated *miR-9a* or a scrambled *miRNA,* we isolated the biotin–miRNA–RISC–mRNA complex with streptavidin beads. Similar to the known *miR-9a/miR-9* targets *senseless*^13^ and *Foxg1* (ref. 14), we found that *miR-9a* binds to and enriches *sNPFR1* mRNA over *RP49* control RNA in *Drosophila* S2 cells and *NPY2R* mRNA over *GAPDH* control RNA in rat insulinoma INS-1 cells (Fig. 6b,e). Consequently, *miR-9a* transfection reduces sNPFR1 and NPY2R protein levels compared with an actin control in both *Drosophila* S2 and rat insulinoma cells, respectively (Fig. 6c,f). In addition, *miR-9a*^*−/−*^ null mutant flies produce significantly more sNPFR1 protein than the wild-type control strain (Supplementary Fig. 7). These results support the hypothesis that the *sNPFR1* and *NPY2R* mRNAs are legitimate targets of *miR-9a/miR-9*.

**Figure 7:**
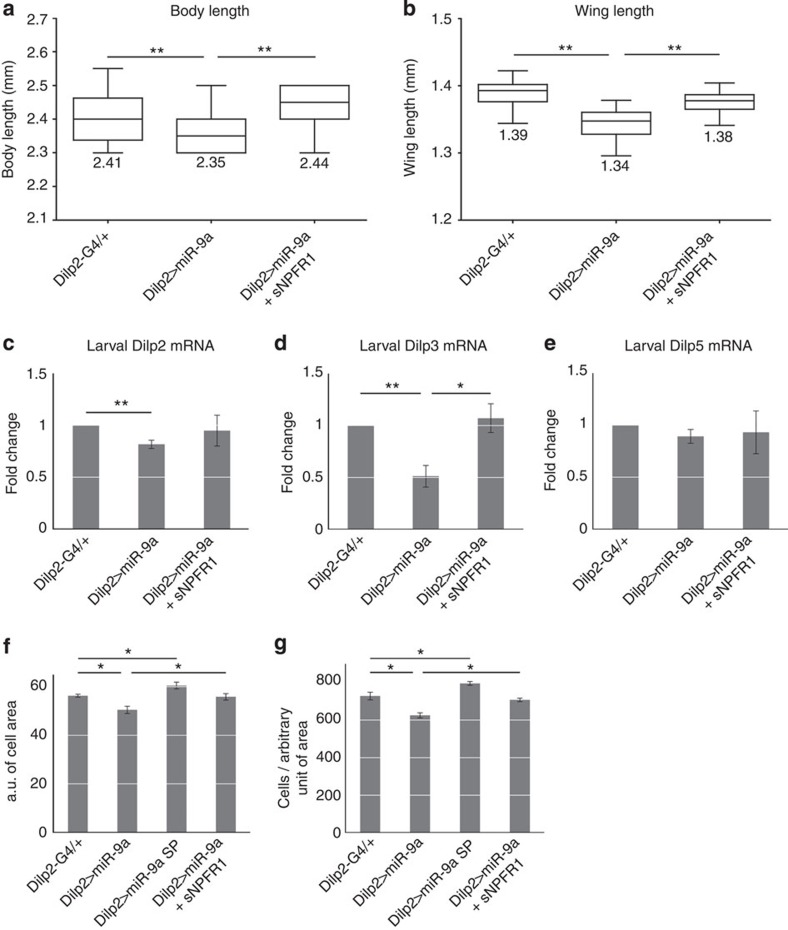
Simultaneous *sNPFR1* overexpression rescues the phenotypes induced by *miR-9a* overexpression. (**a**,**b**) Boxplots indicating that the reduced body and wing length phenotypes induced by IPC-specific *miR-9a* overexpression (*Dilp2>miR-9a*) are rescued by simultaneous overexpression of the *miR-9a* target *sNPFR1* (*Dilp2>miR-9a+sNPFR1)*. (**c**–**e**) The reduction of *Dilp2* and *3* observed in *Dilp2>miR-9a* flies is rescued in *Dilp2>miR-9a+sNPFR1* flies. (**f**) The wing growth defect induced by IPC-specific *miR-9a* overexpression (*Dilp2>miR-9a*) stems from a reduction in both wing cell size and number. This defect can be rescued by simultaneous *sNPFR1* overexpression (*Dilp2>miR-9a+sNPFR1*). (**g**) The increase in wing size observed in the *Dilp2>miR-9a sponge* flies stems from an increase in both cell size and number. Data in **c**–**g** are presented as mean±s.e.m. from at least three independent experiments. Statistical significance was assessed by two-tailed Student's *t*-test, *P<0.05, **P<0.01.

**Figure 8:**
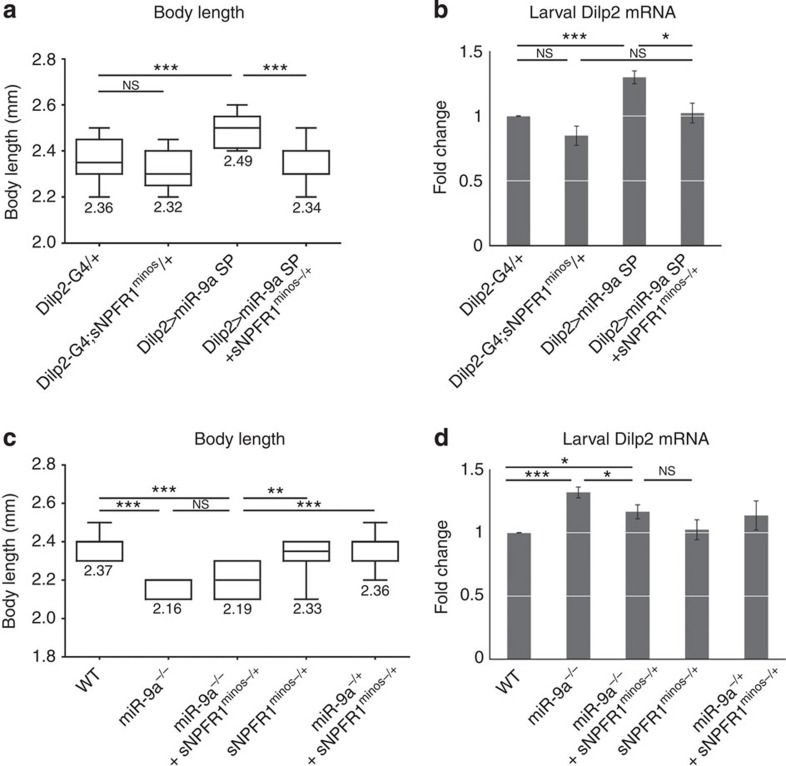
***sNPFR1*** **heterozygous rescues the body length and** ***Dilp2*** **expression by IPC-specific knockdown of** ***miR-9a***. (**a**,**b**) Addition of *sNPFR1*^*minos−/+*^ heterozygosity rescues the body length and *Dilp2* expression phenotype caused by *miR-9a* knockdown in the IPCs (*Dilp2>miR-9a sponge+sNPFR1*^*minos−/+*^). Compare with the *Dilp2>miR-9a sponge, Dilp2-Gal4/+* and *Dilp2-Gal4; sNPFR1*^*minos−/+*^ controls. (**c**,**d**) *sNPFR1*^*minos−/+*^ heterozygosity also rescues body length and *Dilp2* expression in the *miR-9a*^*E39/J22*^ null mutants background (*miR9a*^*−/−*^*+sNPFR1*^*minos−/+*^) compared with *miR-9a*^*E39/J22*^ null mutants. *WT, sNPFR1*^*minos−/+*^ and *miR9a*^*−/+*^*+sNPFR1*^*minos−/+*^ are the controls. Data are presented as mean±s.e.m. from at least three independent experiments. Statistical significance was assessed by two-tailed Student's *t*-test, NS, not significant, *P<0.05, **P<0.01, ***P<0.001.

**Figure 9:**
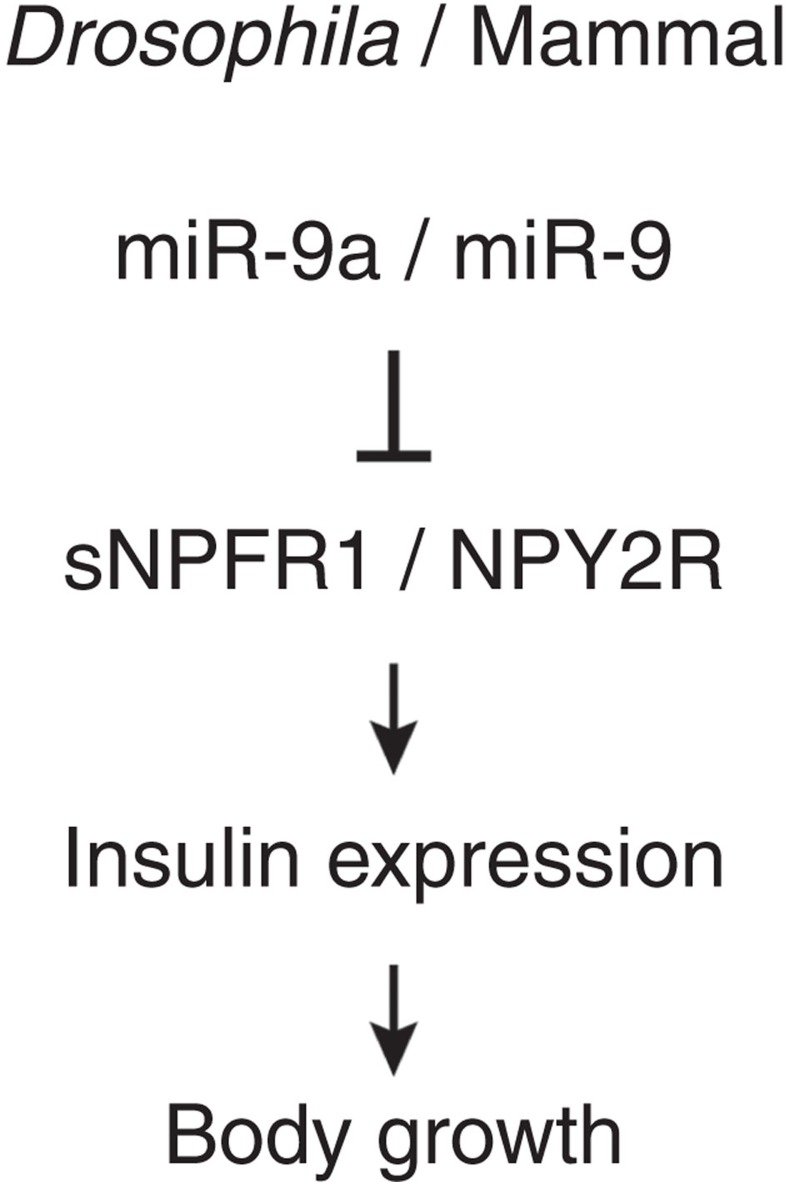
A model summarizing the results of this study. .

To further support our hypothesis that *miR-9a* inhibits growth and suppresses insulin signalling via its target *sNPFR1*, we simultaneously overexpressed both *miR-9a* and *sNPFR1* in the *Drosophila* IPCs. Indeed, overexpression of *sNPFR1* rescues the reduced body and wing length phenotypes of *Dilp2>miR-9a* flies (Fig. 7a,b). Similarly, *sNPFR1* overexpression also restores *Dilp2* and *3* expression levels (Fig. 7c–e). We next asked whether reduced *sNPFR1* dosage (*sNPFR1*^*minos−/+*^ heterozygote) can restore body length and *Dilp2* expression in the background of *miR-9a* knockdown via *miR-9a sponge*. The *sNPFR1* heterozygote restores body length significantly in the *miR-9a sponge* (*Dilp2>miR-9a sponge+sNPFR1*^*minos−/+*^) and slightly in the *miR-9a*^*−/−*^ null mutants (*miR-9a*^*−/−*^*+sNPFR1*^*minos−/+*^) (Fig. 8a,c). The *sNPFR1* heterozygote also restores *Dilp2* expression in the *miR-9a* sponge and *miR-9a*^*−/−*^ null mutant backgrounds (*Dilp2>miR-9a sponge+sNPFR1*^*minos−/+*^or *miR-9a*^*−/−*^*+sNPFR1*^*minos−/+*^) (Fig. 8b,d). Finally, we further characterized the wing growth phenotype caused by *miR-9a* manipulation by measuring both cell size and cell number. Compared with the control (*Dilp2-Gal4/+*), overexpression of *miR-9a* in the IPCs (*Dilp2>miR-9a)* reduces both wing cell size and number, while knockdown of *miR-9a* (*Dilp2>miR-9a sponge*) increases wing cell size and number. As expected, the changes in wing cell size and number induced by *miR-9a* overexpression in the IPCs are rescued by simultaneous overexpression of *sNPFR1* (*Dilp2>miR-9a+sNPFR1*) (Fig. 7f,g). These results confirm that *miR-9a* in IPCs regulates insulin signalling and body growth via its target *sNPFR1* (Fig. 9).

## Discussion

We have uncovered a novel function for *miR-9a* in the regulation of insulin signalling and body growth through its target *sNPFR1* in the *Drosophila* brain IPCs. In a *miRNA* overexpression screen using the IPC driver *Dilp2-Gal4*, we found that *miR-9a* and the closely related *miR-9b* dramatically reduce wing size (Fig. 1). We were able to verify that *miR-9a* is endogenously expressed in the larval and adult IPCs via LNA *in situ* hybridization against the mature miRNA and via *miR-9a* sensor lines (Fig. 2; Supplementary Fig. 2). IPC-specific overexpression of *miR-9a* also reduces body size, while knockdown of *miR-9a* via expression of a *miR-9a sponge* increases body size. This effect is likely caused by modulation of insulin expression, as *miR-9a* overexpression reduces *Dilp2* levels, while *miR-9a* knockdown enhances *Dilp2* levels (Fig. 3).

Contrary to expectations, we found that while sponge-mediated knockdown of *miR-9a* in the IPCs increases body size, *miR-9a*^*−/−*^ null mutants and flies in which *miR-9a* is suppressed in non-IPCs show reduced body size. In other words, loss of *miR-9a* in the IPCs alone increases growth while ubiquitous or non-IPC loss of *miR-9a* reduces growth (Figs 3a, 4a and 5b). We found, however, that the level of *Dilp2* mRNA is increased in both *Dilp2>miR-9a sponge* and *miR-9a*^*−/−*^ null mutant flies and the level of the downstream signalling protein pAKT is significantly increased in the *miR-9a* mutants (Figs 3d,g and 4c,f). This activation of insulin signalling in both the *miR-9a sponge* flies and the *miR-9a*^*−/−*^ null mutants suggests that *miR-9a* has positive effects on growth in non-IPCs that are able to mask its negative effect on growth in the IPCs.

In the IPCs, *miR-9a* is likely mediating its effects by targeting the known positive regulator of insulin expression *sNPFR1* as *miR-9a*^*−/−*^ null mutants express elevated levels of sNPFR1 (Supplementary Fig. 7). Mammals have multiple NPY receptors, but sNPFR1 is strongly similar to NPY2R (ref. 7). The germ-line deletion of *NPY2R* reduces mouse body weight and adiposity^15^ and the *sNPFR1* dominant negative mutation reduces fly body growth through ERK^5^. Although the IPCs of invertebrates are localized in the brain and the IPCs of mammals are in the pancreas, the functional similarities of the two systems are clear. *sNPF* overexpression in flies causes hyperinsulinemia and enhances body growth^5^, while NPY overexpression in mammals causes hyperinsulinemia, hyperphagia and obesity^16^. In mice, a triple knockout of NPY1R, NPY2R and NPY4R prevents the hyperinsulinemia associated with NPY overexpression, indicating that NPY signalling through the NPY receptors modulates insulin secretion^17^.

Here we show that both fly *sNPFR1* and mammalian *NPY2R* are targets of *miR-9a/miR-9* by demonstrating direct binding of *miR-9a/miR-9* to the *sNPFR1* and *NPY2R* 3′-UTRs using an *in vitro* binding assay and a CLIP assay (Fig. 6). Our results suggest an evolutionarily conserved relationship between the *miR-9* family and the sNPF/NPY receptors (Fig. 9). *miR-9* in mammals is expressed in both the brain and the pancreatic beta cells where it has been shown to regulate glucose levels via its targets *onecut2* and *sirt1* (refs 18, 19). In addition to its effects in the brain on pancreatic insulin secretion, NPY is also released by the autonomic nervous system into the pancreas where it directly modulates insulin and glucagon secretion^20^. It will thus be interesting to see if the relationship we have uncovered between the *miR-9*, NPY, insulin signalling and body growth extends to *in vivo* mammalian models despite the differences in anatomical location and embryonic origin of the IPCs.

## Methods

### *Drosophila* culture and stocks

All flies were maintained at 25 °C on standard cornmeal, yeast, sugar, agar medium. *Wild-type Oregon R, w*^*1118*^ and *sNPFR1*^*minos*^ were obtained from the Bloomington Stock Center (Bloomington, USA). *Dilp2-Gal4* was provided by E. Rulifson (University of California San Francisco, USA), and *miR-9a*^*J22*^ and *miR-9a*^*E39*^ mutants were provided by F.B. Gao (University of Massachusetts Medical School, USA). *miR-9a sponges* were provided by D. Van Vactor (Havard medical school, USA). *Tubulin–GFP–miR-9a* sensor was provided by E. Lai (Sloan-Kettering Institute, USA). The *UAS-sNPFR1* transgenic fly was generated by the p-element-mediated germline transformation method with the *pUAS–sNPFR1* construct containing the full length of *sNPFR1* cDNA in the pUAS vector^5^. To generate the *Dilp2-Gal80*^*ts*^ line, we amplified the *Dilp2* promoter (Supplementary Table 4 for primer sequences) via PCR and inserted it into pBPGAL80Uw-6 (a gift from Y.-J.K.). We then microinjected the resulting construct for site-specific insertion on chromosome 2L (PBac{y+-attP-3B}VK00037 embryos) using standard methods.

### *UAS-miRNA* library construction

We obtained 45 previously published *UAS-DsRed-pre-miRNA* constructs from the *Drosophila* RNAi Screening Center^21^. Using overlapping oligos, we modified these vectors by adding minimal attB sites into the Mlul site to make them suitable for site-specific insertion into the fly genome. To produce the rest of the stocks, we modified an attB-containing *SST13 UAS* vector^22^, which makes use of the split-white system to reduce time spent on off-target insertions^23^. We inserted additional restriction sites between the KpnI and XbaI sites of the *SST13* polylinker using overlapping oligos (5′-*CTAGAGCGAGCTCAAGGCCACTAGGGCCGGGATCGATATGGTAC*-3′ and 5′-*CATATCGATCCCGGCCCTAGTGGCCTTGAGCTCGCT*-3′). Inspired by the design of the Silver *et al.*^21^ UAS-miRNA vectors, we also inserted the DsRed coding sequence downstream of a 5 × UAS sequence and renamed this vector *pSS-DsRed*. Next, we amplified putative pri-miRNA sequences consisting of roughly 400–500 bp surrounding each mature miRNA sequence and inserted them into *pSS-DsRed*. We validated all the *UAS-miRNA* vectors with restriction digests and sequencing. See Supplementary Table 1 for a summary of these *UAS-miRNA* constructs and the primers used to make them. All of our *UAS-miRNA* constructs were inserted into a specific attP site on the second (VIE-72a, a gift from B. Dickson) or third chromosome (VIE-49b, a gift from B. Dickson) using the ФC31 integrase system and standard transgenesis techniques.

### miRNA *in situ* hybridization

We performed the miRNA *in situ* hybridizations on larval and adult *Drosophila* brains essentially by the standard technique^24^ with minor modifications as follows: We fixed the brains for 1 h at room temperature and used methanol for washing instead of ethanol. We did not use any Proteinase K treatment, nor did we do any secondary fixation. We used a DIG-labelled miR-9a-specific LNA probe purchased from Exiqon (#88078-15) at 50 nM for larval brains and 250 nM for adult brains. We performed the hybridization reaction and subsequent washings at 60 °C for larval brains and 65 °C for adult brains. We visualized binding of the LNA probe with an anti-DIG antibody coupled to alkaline phosphatase, developing the larval brains for 80 min and the adult brains for 2 days. The elevated concentration and development time required for the adult *in situ* may indicate either lower expression or some degree of probe degradation, but the staining was clearly specific as the *miR-9a* null mutant brains stained at the same time and under the same conditions were blank.

### Cell culture and transfection

We purchased *Drosophila* S2 cells from the *Drosophila* Genomics Resource Center (DGRC, the Indiana University) and maintained them at 26 °C in Schneider media supplemented with 10% bovine calf serum. We cultured rat insulinoma INS-1 cells in 5% CO_2_ at 37 °C in RPMI 1640 media containing 2 mM L-glutamine supplemented with 10% foetal bovine serum, 5.6 mM glucose, 10 mM HEPES, 1 mM sodium pyruvate, 50 μM 2-mercaptoethanol, 100 IU ml^−1^ penicillin and 100 μM streptomycin. We transfected the cells with either synthetic scrambled *miR-9a* or *miR-9a* duplex (Bioneer, Korea) (final concentration 80 μM) using Xtremegene HP (Roche, USA). The scrambled *miR-9a* and *miR-9a* duplex sequences used in the transfection are listed in Supplementary Table 3.

### CLIP assay and the miRNA–mRNA pull-down assay

We performed the CLIP assay^11^,^25^ with the following modifications: 24 h post transfection, we irradiated the cells with 150 mJ cm^−2^ at 254 nm using a CL-100s UV Cross-linker. We then harvested the cells, added lysis buffer (Cell Signaling, USA) and treated them with a low dose of RNase A (100 ng ml^−1^) for partial RNA digestion. We then used Protein A Dynabeads (Invitrogen, USA) and 2 μg AGO-1-specific antibody for the immuno-precipitation. The immunoprecipitate was treated with 20 μg ml^−1^ proteinase K for 10 min at 37 °C. We then extracted the RNA using the easy-BLUE kit (iNTRON, Korea) and synthesized cDNAs using the SuperScript III First-Strand Synthesis System (Invitrogen). To determine whether *miR-9a* directly binds to *sNPFR1* and *NPY2R* mRNA, we designed primers to amplify fragments of their 3′-UTRs that include the predicted *miR-9a* seed sequence matches (Supplementary Table 4). We used standard PCR and the Zero Blunt TOPO PCR Cloning Kit (Invitrogen) to obtain and then sequence several clones for each condition (Supplementary Table 5).

We performed the biotin–RNA pull-down assay^26^ with the following modifications: The cells were harvested after transfection for 24 h and lysed in lysis buffer (Cell Signaling) containing 20 × protease inhibitor (Roche) and 60 U RNaseOUT (Invitrogen). We used streptavidin agarose beads (GE Healthcare, UK) for the immuno-precipitation. We extracted the RNA using the easy-BLUE kit and synthesized cDNAs using the SuperScript III First-Strand Synthesis System. We then performed quantitative real-time PCR using primers (Supplementary Tables 3 and 4) specific to fragments of the 3′-UTRs of *sNPFR1* and *NPY2R* that contain the *miR-9a* seed match. We used *senseless* and *foxg1* as positive controls and *actin* and *tubulin* as negative controls.

### Quantitative RT–PCR analysis

We collected heads or bodies from 15 adult flies and isolated total RNA with the easy-BLUE reagent. After treating the RNA samples with RNase-free DNase I (TAKARA, Japan), we synthesized cDNA using the SuperScript III First-Strand Synthesis System. We then performed quantitative reverse transcription–PCR (RT–PCR) analysis with the StepOnePlus Sequence Detection System (Applied Bio Systems, USA) using the SYBR Green PCR Core reagents (Applied Bio Systems). We performed each experiment at least three times and used the comparative cycle threshold to present a fold change for each specific mRNA after normalizing to *rp49* levels. The primers we used in our qPCR analyses are listed in Supplementary Table 3.

### Fluorescent western blots

We isolated total protein from the bodies of adult flies or larvae and from *Drosophila* S2 cells or rat INS-1 cells with the PROPREP protein extraction buffer (iNtRON) or RIPA buffer (Cell Signaling). We subjected 30 μg of the fly proteins and 80 μg of the cell line proteins to SDS–PAGE and transferred them to low fluorescence PVDF membrane (Thermoscientific, USA). We blocked the membranes for 60 min in TBS buffer containing 5% BSA and incubated them overnight at 4 °C with primary antibodies. After incubation in the dark with fluorophore-tagged secondary antibodies and subsequent washes, we digitized images of the membranes using the Amersham Imager 600 (Amersham Biosciences, USA). We then quantified target protein versus their internal loading control band intensities with the Tina2.0 software (UK). We used the following primary antibodies: anti-phospho-Akt (1:1,000, Cell Signaling), anti-Akt (1:1,000, Cell Signaling), anti-NPY2R (1:1,000, Santa Cruz, USA), anti-β-actin for *Drosophila* (1:1,000, Developmental Studies Hybridoma Bank, DSHB, USA) and anti-β-actin for mammals (1:1,000, Santa Cruz). We used the following secondary antibodies: Goat anti-rat IgG, Dylight488 (1:1,000, Pierce, USA), Goat anti-mouse IgG, DyLight488 (1:1,000, Thermoscientific), Goat anti-rabbit IgG, DyLight488 (1:1,000, Thermoscientific) and Alexa fluor 680 donkey anti-rabbit IgG (1:500, Life technologies, USA) (Supplementary Fig. 8).

### Measurement of body length and wing length

To avoid artifacts caused by vial over-crowding, we maintained population densities of 50 embryos per vial in standard fly media. Using the Image J analysis package and a microruler, we measured the body length of adult males 3–5 days post eclosion from the anterior end of the head to the posterior end of the abdomen. We measured wing length from the ACV to the end of third longitudinal vein (L3).

### Measurement of cell size and cell number

To quantify relative cell size and cell number, wings were digitally photographed at × 200 magnification. Image J analysis software was used to measure relative pixel areas of wings, using wing vein landmarks to retain fair morphological comparisons. Adobe Photoshop software was used to measure a 100 × 100 pixel area of the L4–L5 inter-vein region. We counted epidermal hairs within this same pixel area to measure relative pixel area per cell or cell size. To calculate relative cell number, the wing pixel area was multiplied by the relative pixel area per cell. Data were collected for males only.

### Measurement of the haemolymph glucose and trehalose levels

We collected 3–5 days–old-adult flies to measure glucose and trehalose levels in haemolymph. To collect the haemolymph, the flies were punched in the thorax and then transferred to 0.5 ml tube placing in a 1.5 ml collection tube. The tubes were then centrifuged at 5,000 r.p.m. for 5 min. About 1 μl of haemolymph was added to 30 μl of 1 × trehalose solution (Sigma, USA) and incubated for 16–18 h at 37 °C. Next, we added Glucose assay (GO) reagent (Sigma) and incubated at 37 °C for 30 min. The reaction was stopped by the addition of 12 N H_2_SO_4_. Total haemolymph glucose concentration (from trehalose and glucose) was measured using microplate reader (BioTek, USA) at 540 nm. Standard curves were generated by D-(+)-glucose solution (Sigma) for every experimental trial. A linear regression of concentration samples over the standard range was used to find actual concentration from haemolymph samples.

### Statistics

Boxplots in this study follow the standard style except that the whiskers represent minimum to maximum. Regarding the bar charts, unless otherwise noted, we present the mean±s.e.m. and make comparisons between groups using the Student's *t*-test considering *P*<0.05 as statistically significant. For the multiple comparisons made in Fig. 1a, we performed a one-way analysis of variance followed by pairwise *t*-tests using the Bonferroni method to adjust the *P* value threshold for significance.

## Additional information

**How to cite this article:** Suh, Y. S. *et al.* Genome-wide microRNA screening reveals that the evolutionary conserved miR-9a regulates body growth by targeting sNPFR1/NPYR. *Nat. Commun.* 6:7693 doi: 10.1038/ncomms8693 (2015).

## Supplementary Material

Supplementary Information Supplementary Figures 1-8 and Supplementary Tables 1-5
