# Supplementary material for: Genome-wide microRNA screening reveals that the evolutionary conserved miR-9a regulates body growth by targeting sNPFR1/NPYR
Source: Nat Commun. 2015 Jul 3;6:7693. doi: 10.1038/ncomms8693 (PMC4506552; doi:10.1038/ncomms8693)
Supplement: Supplementary Information — Supplementary Figures 1-8 and Supplementary Tables 1-5 [file ncomms8693-s1.pdf]

Supplementary Figure 1. Vector schematic for *UAS-miRNA* generation.

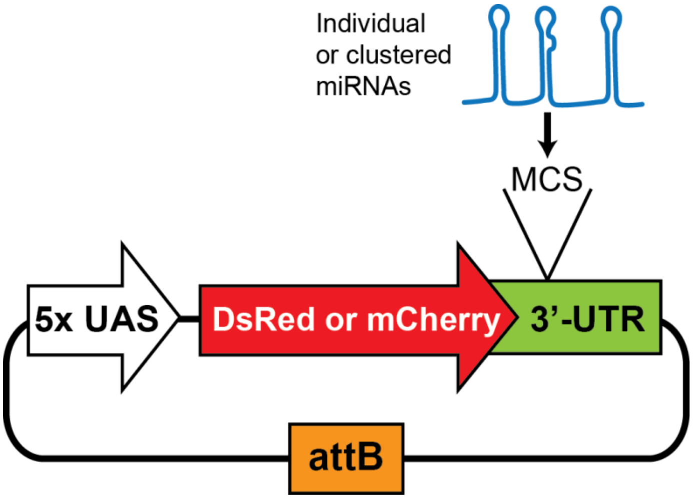

All *miRNAs* (clustered and individual) were inserted into a multiple cloning site in the 3'-UTR of a fluorescent marker.

Supplementary Figure 2. *miR-9a* is expressed in the IPCs of the larval brain

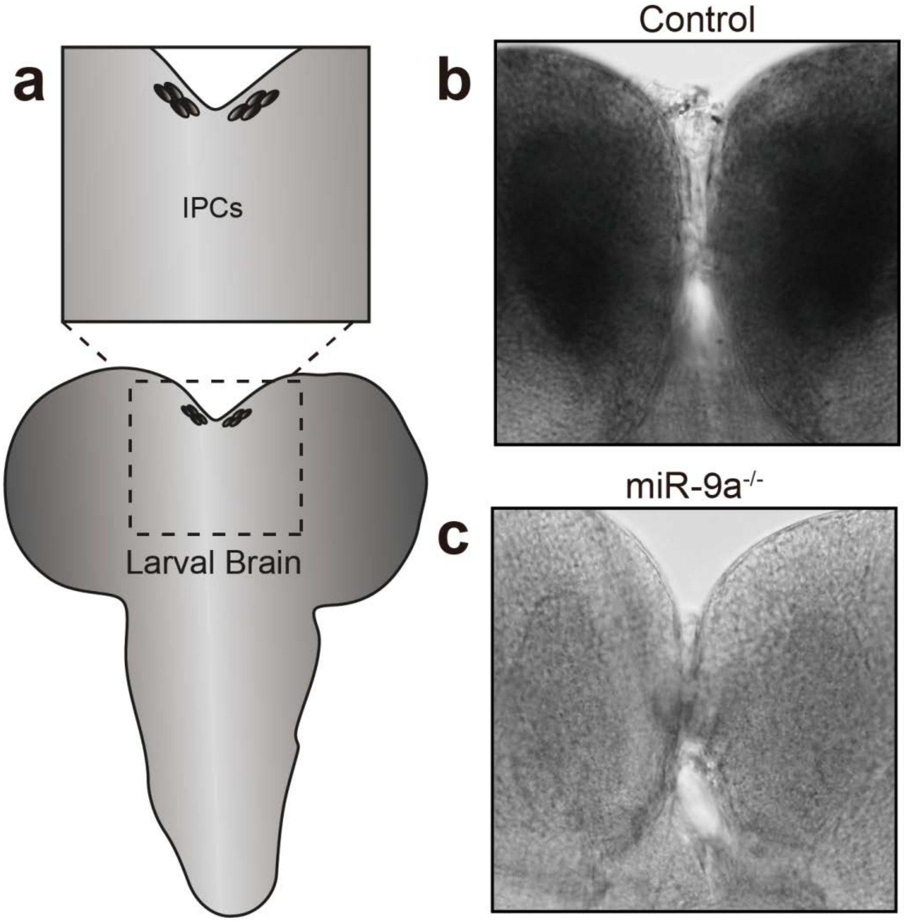

(a) Larval brain schematics indicate the position of the IPCs in the median neurosecretory cluster in the *pars intercerebralis.* An *in situ* hybridization with an LNA probe specific to the mature *miR-9a* sequence stains the IPCs in the w^{1118} control genotype (b), but not in $miR\text{-}9a^{E39/J22}$ *null mutant* ($miR\text{-}9a^{-/-}$) brains (c).

Supplementary Figure 3. The effect of *miR-9a* on pupal volume

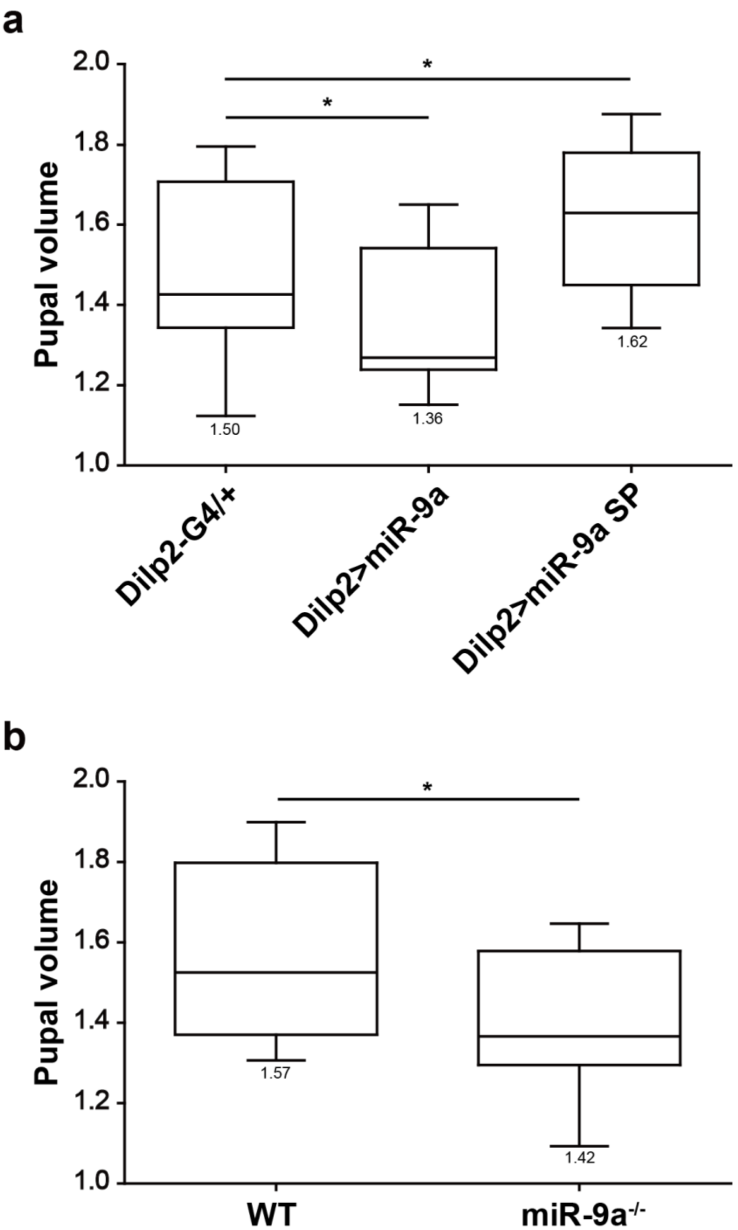

(a) IPC-specific *miR-9a* over-expression (*Dilp2>miR-9a*) reduces pupal volume, while IPC-specific *miR-9a* knockdown (*Dilp2>miR-9a sponge*) increases pupal volume as compared to *Dilp2-Gal4/+* control flies. (b) *miR-9a*$^{E39/J22}$ null mutants (*miR-9a*$^{-/-}$) produce smaller pupae than w*ild-type (WT)* controls. Data are presented as mean ± s.e.m. from at least three independent experiments. Statistical significance was assessed by two-tailed Student's *t*-test, **P*<0.05.

Supplementary Figure 4. The effect of *miR-9a* on *Dilp* levels in adult heads.

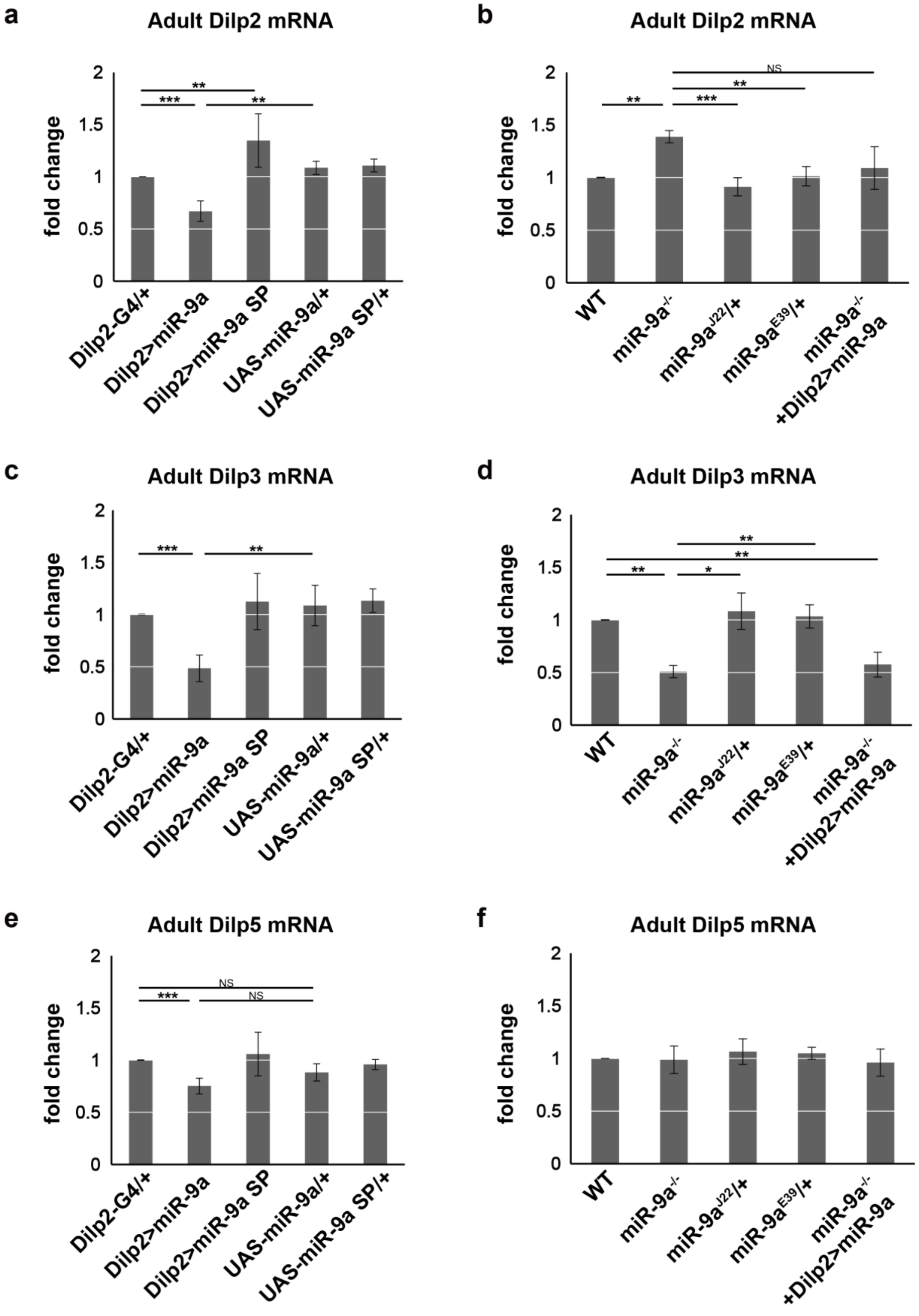

(a, c, e) IPC-specific *miR-9a* over-expression (*Dilp2>miR-9a*) reduces *Dilp2, 3, and 5* mRNA levels, while IPC-specific *miR-9a* knockdown (*Dilp2>miR-9a sponge*) increases the level of *Dilp2* mRNA compared to *Dilp2-Gal4/+*, *UAS-miR-9a/+*, *UAS-miR-9a sponge/+* controls. (b, d, f) *miR9a*$^{E39/J22}$ null mutants increase the level of *Dilp2* mRNA and decrease the level of *Dilp3* mRNA compared to *WT, miR-9a*^{J22}*/+,* and *miR-9a*^{E39}/+ heterozygous controls. Data are presented as mean ± s.e.m. from at least three independent experiments. Statistical significance was assessed by two-tailed Student's *t*-test, NS: not significant, * *P*<0.05, ** *P*<0.01, *** *P*<0.001.

Supplementary Figure 5. The effect of *miR-9a* on activated pAKT in adult bodies.

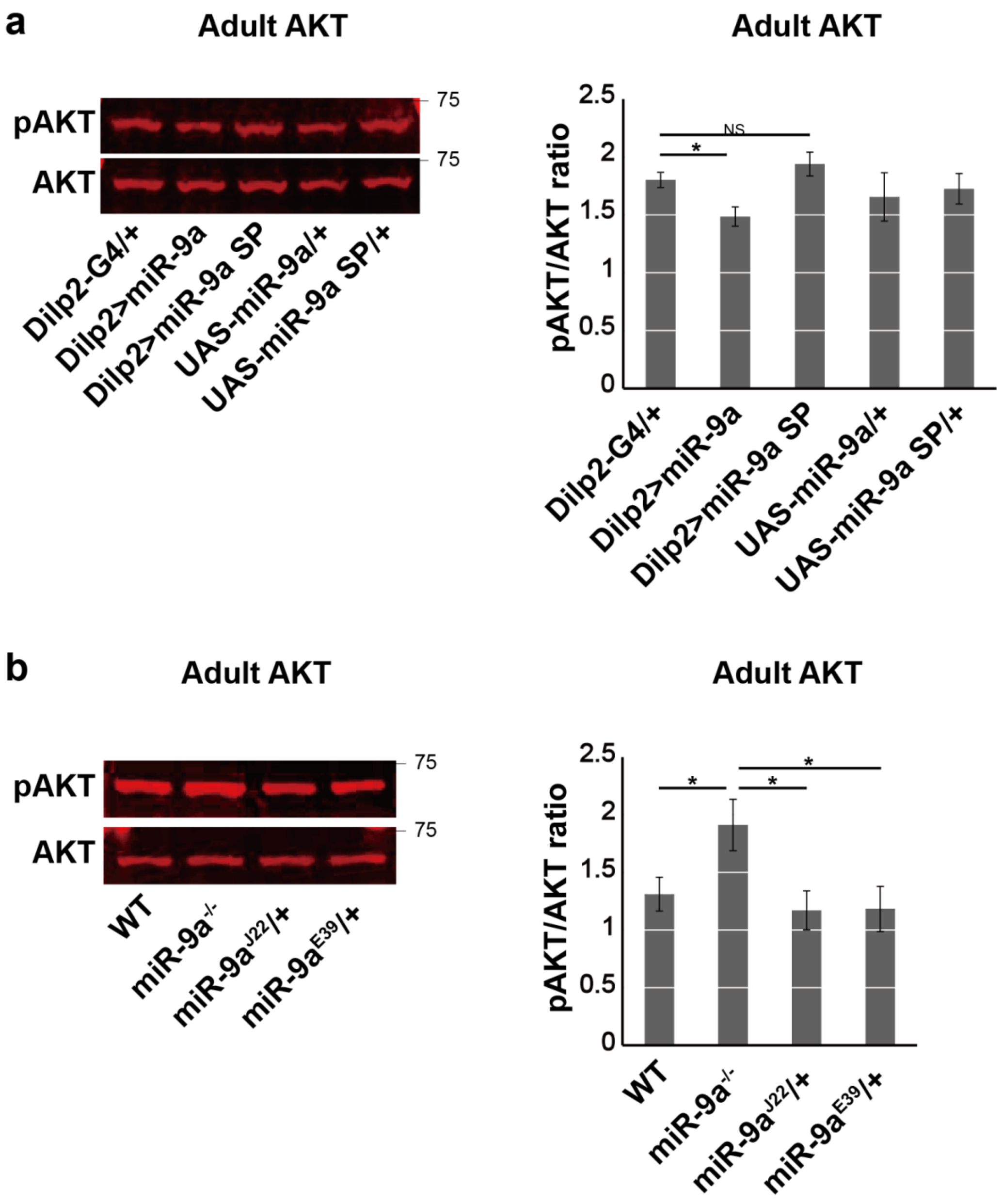

(a) IPC-specific *miR-9a* over-expression (*Dilp2>miR-9a*) reduces activated pAKT compared to *Dilp2-Gal4/+* and *UAS-miR-9a/+* controls. (b) *miR-9a*$^{E39/J22}$ null mutants show elevated activated pAKT compared to *WT, miR-9a*^{J22}/+, and *miR-9a*^{E39}/+ heterozygote controls. Data are presented as mean ± s.e.m. from at least three independent experiments. Statistical significance was assessed by two-tailed Student's *t*-test, NS: not significant, * *P*<0.05.

Supplementary Figure 6. The effect of *miR-9a* on glucose and trehalose levels.

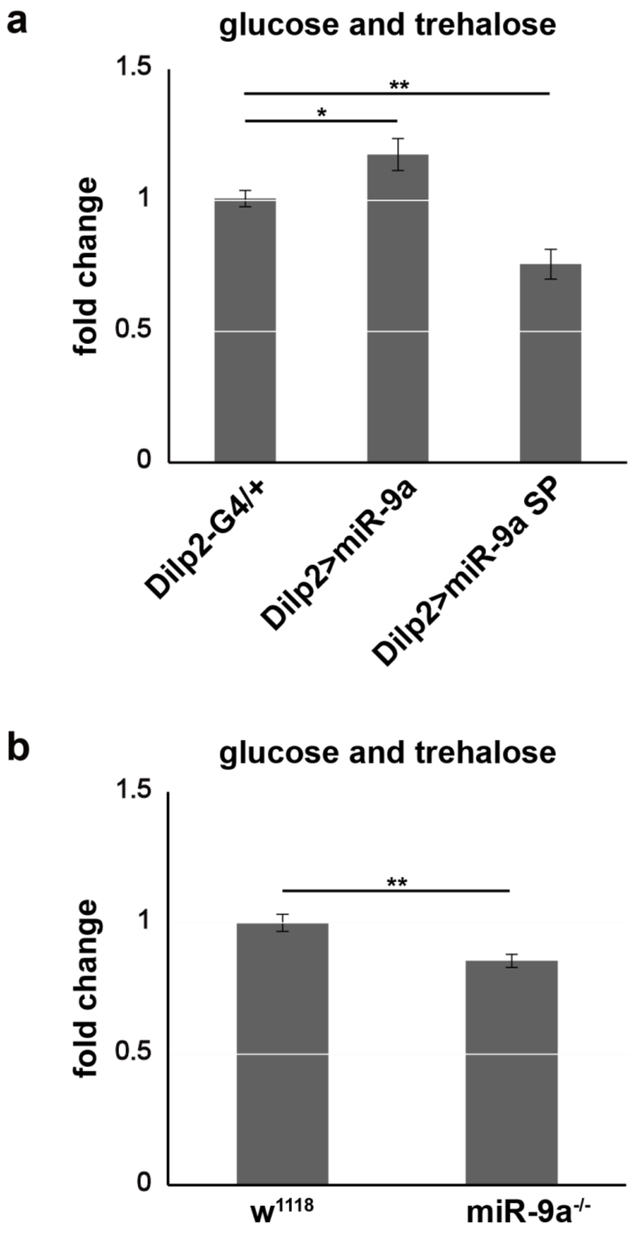

(a) IPC-specific *miR-9a* over-expression (*Dilp2>miR-9a*) increases the level of glucose and trehalose in the hemolymph, while IPC-specific *miR-9a* knockdown (*Dilp2>miR-9a sponge*) reduces the level of glucose and trehalose in the hemolymph compared to *Dilp2-Gal4/+* controls. (b) $miR\text{-}9a^{E39/J22}$ null mutants show a reduced level of hemolymph glucose and trehalose when compared to w^{1118} control flies. Data are presented as mean ± s.e.m. from at least three independent experiments. Statistical significance was assessed by two-tailed Student's *t*-test, * $P<0.05$, ** $P<0.01$.

Supplementary Figure 7. sNPFR1 protein is elevated in *miR9a*$^{-/-}$ null mutants.

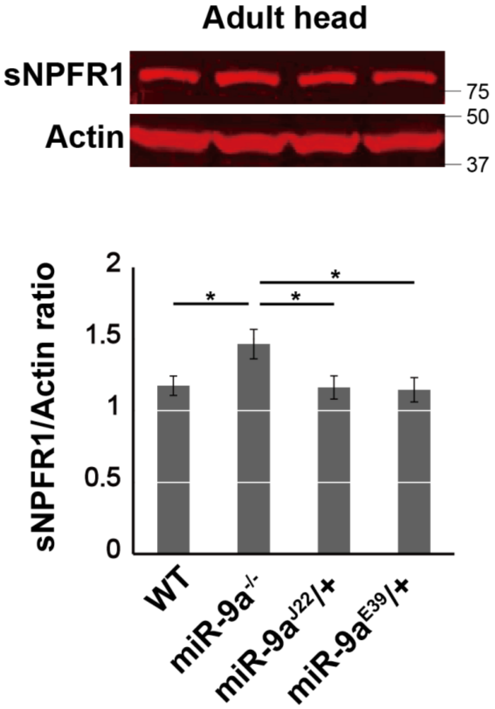

sNPFR1 protein is elevated in *miR9a*$^{E39/J22}$ null mutants compared with *WT*,*miR-9a*^{J22}/+, *miR-9a*^{E39}/+ heterozyous controls. Data are presented as mean ± s.e.m. from at least three independent experiments. Statistical significance was assessed by two-tailed Student's *t*-test, * P<0.05.

Supplementary Figure 8.   Images of blot used in this study.

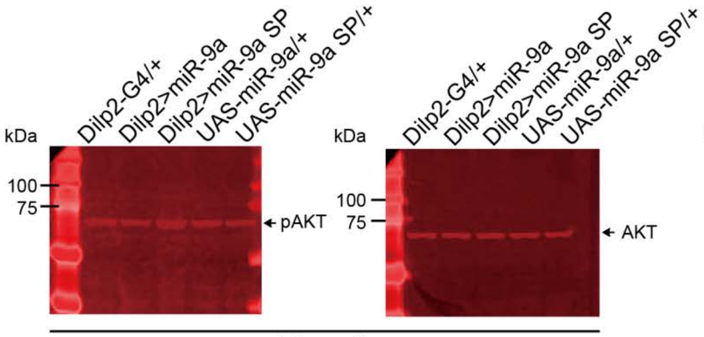

Figure 3g

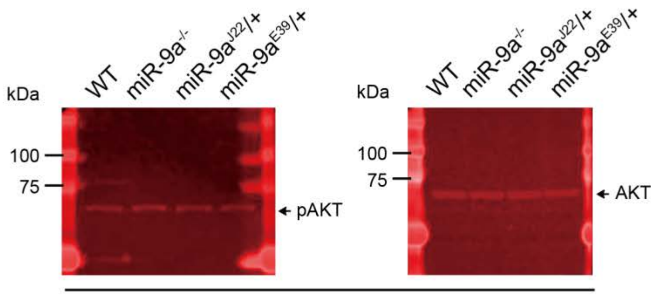

Figure 4f

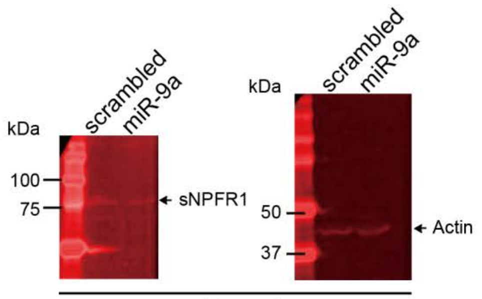

Figure 6c

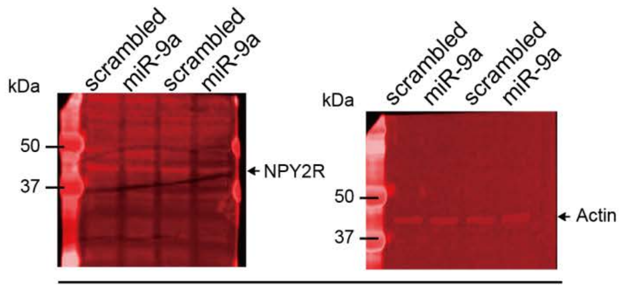

Figure 6f

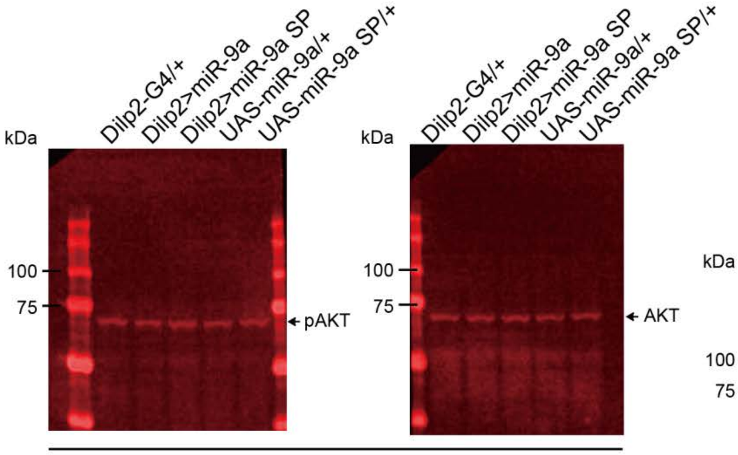

Supplementary Figure 5a

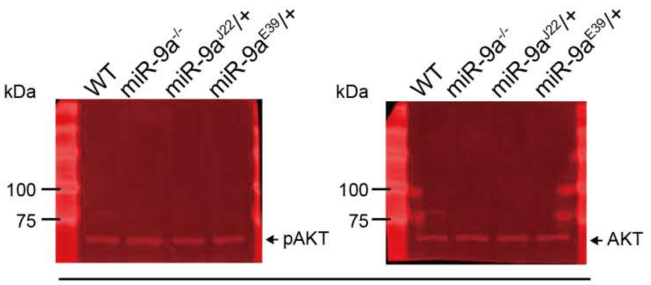

Supplementary Figure 5b

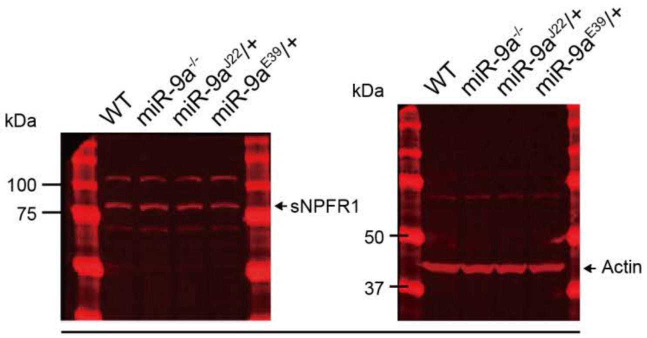

Supplementary Figure 7

Supplementary Table 1. Primers used to generate the *UAS-miRNA* stocks

miRNA	Vector	Primers (5' to 3')
let-7	*pSS-DsRed*	F: CCGGGTACCAGATCAACAGCGATCCATTAAACA<br>R: CCGTCTAGAAGTGGTGCAGTTCGATTGGGA
bantam	*pSS-DsRed*	F: CCGGGTACCTCTCGTTCTTCGCTTCTCTGTGGT<br>R: CCGTCTAGAACCTTCGATTTCCCAGCCCCA
miR-2a-2	*pSS-DsRed*	F: CCGGGTACCGGGGGACAGATGCATGCCACA<br>R: CCGTCTAGATGGCCAGTAGTCTCCAAGCACCA
miR-2a-1	*pSS-DsRed*	F: CCGGGTACCGACGCGATGCTCAAGGCAAAAA<br>R: CCGTCTAGAGTGGCATGCATCTGTCCCCCG
miR-2b-2	*pSS-DsRed*	F: CCGGGTACCTCGTGTTGTAGTCTCGTCGTCGC<br>R: CCGTCTAGAAGCCCAGCATGAATGCGCCA
mir-3/309	*pSS-DsRed*	F: CCGGGTACCATTCGCGGAACAGCCCCGAC<br>R: CCGTCTAGAAGAGACCATACCGACATTCGCCA
miR-4/5/286	*pSS-DsRed*	F: CCGGGTACCGCCACATCGTCGCAACTTCAAATCAA<br>R: CCGTCTAGAACAGCACAGCAACTATTCCCTCTACA
miR-6-1/6-2/6-3	*pSS-DsRed*	F: CCGGGTACCAGGAAAATGAAAGTCAAAGTTGGCAGC<br>R: CCGTCTAGAAACCAACGTCTCATCAGTTTTTCCCA
miR-7	*pSS-DsRed*	F: CCGGGTACCACGCGTGATTAATTTGGAAGGAAAGGT<br>R: CCGTCTAGAGAGTTGGCGGCAGGATGGCA
miR-8	*pSS-DsRed*	F: CCGGGTACCTCGCCAAGGGGGGCCAATGTT<br>R: CCGTCTAGAAAAACGCGTCGTTGTGCCGC
miR-11	*pSS-DsRed*	F: CCGGGTACCGTGTCACGCTGCAGGGCCTC<br>R: CCGTCTAGACAATTTTGAACTGATTCTCGTTGGCCG
miR-2c/13a/13b-1	*pSS-DsRed*	F: CCGGGTACCTGTGATGGTGTGTGTGCGTCG<br>R: CCGTCTAGATCGACGAGTGCAGGATAGATCCGTT
miR-13b-2	*pSS-DsRed*	F: CCGTCTAGAGGTCGGCGATCTTGGCCAGC<br>R: CCGGGTACCGCTCCGGCGAGCTCAAGTCC
miR-14	*pSS-DsRed*	F: CCGGGTACCGCCGTGGTTTCGCTTCGTGC<br>R: CCGTCTAGATCGGTCGAATGGCTGCTGCT
miR-31a	*pSS-DsRed*	F: CCGGGTACCACTCGAGGCAGCTAAGCGAA<br>R: CCGTCTAGACAAAAACTGTTCCACATTTTGCGAAT
miR-34	*pSS-DsRed*	F: CCGGGTACCGCCAGAAGGTGGCGCTCGAT<br>R: CCGTCTAGAGCGTCTGGTTGAGCAGTTGCC
miR-100	*pSS-DsRed*	F: CCGGGTACCTCGGACAACAGAGCTTGCCC<br>R: CCGTCTAGATGTTTAATGGATCGCTGTTGATCTTCT
miR-125	*pSS-DsRed*	F: CGGGGTACCCCAGCCATACAAAAGTTGGTGGTGC<br>R: CCGTCTAGACCAATCAATCATAGGCAGTAGAACGGC
miR-133	*pSS-DsRed*	F: CCGGGTACCACCGTTTACAACAAAGGCGCGTA<br>R: CGGTCTAGAACCATATTTTGCATACACTCGCAGGC
miR-137	*pSS-DsRed*	F: CCGGGTACCAGCGAAAGGAATGCCCGCCG<br>R: CCGTCTAGATGTTTGTTACGTGCTATCGCATCC
miR-184	*pSS-DsRed*	F: CCGGGTACCGCTCCTCCGCCAGCTGTTGT<br>R: CCGTCTAGACACACTGAGCAGCCACATGGGA
miR-210	*pSS-DsRed*	F: CCGGGTACCTGGCCGCGTTTTAGCCTGGA<br>R: CCGTCTAGATGGCCACTTTGCGTTTGAAATGCT
miR-281-1	*pSS-DsRed*	F: CCGGGTACCTGCCAAGTGGCGCGCAGTTTGTTGA<br>R: CCGTCTAGATCGCGGATCTGAAAGCGGA
miR-281-2	*pSS-DsRed*	F: CCGGGTACCCGATGCGCTGTGAGGCA<br>R: CCGTCTAGAATCGGGCAGAGCAGCCGAGA
miR-289	*pSS-DsRed*	F: CCGGGTACCCCAGCCAGCCGTTCCAGTCG<br>R: CCGTCTAGAACGTGCTTTTCCCCCTGCCC
miR-306/79/9b	*pSS-DsRed*	F: CCGGGTACCGCAACATGCAGTTTGCAGACAATG<br>R: CCGTCTAGACATTGAGAACAAGTAACGCGAGAA
miR-375	*pSS-DsRed*	F: CCGGGTACCCGTGGGGCCCCGATTCTTGG<br>R: CCGTCTAGAGGGTTCAGACCCACCCCCGA
miR-929	*pSS-DsRed*	F: CCGGGTACCGAGGGAGCGTCAGGAGGCCA<br>R: CCGTCTAGAGCTTCGGCGGCGAGTTCCTC
miR-932	*pSS-DsRed*	F: CCGGGTACCATGTACGGTGGACTACAGAAAATGA<br>R: CCGTCTAGAAGTGGCCGGTGATGGTAATGT
miR-955	*pSS-DsRed*	F: CCGGGTACCTCCGACCAGTTAAAGAAACAAACTACA<br>R: CCGTCTAGATGGCTATTTGAATTGGGCTACAACC
miR-959/960/961/962	*pSS-DsRed*	F: CCGGGTACCTGGATAATGTGCCCAGTGGATGCT<br>R: CCGTCTAGAGGAGTCCTTTGTAGTGTATTGTCTGGT
miR-969	*pSS-DsRed*	F: CCGGGTACCCGCAGTGCCAGCATCTGGGAAA<br>R: CCGTCTAGAACAATGGGAACTAAAGTGCAATAAATG
miR-970	*pSS-DsRed*	F: CCGGGTACCAGCGTTCAGCCCCAAGCCAC<br>R: CCGTCTAGAGCCCGATTTGTCGCCAATCGCTA
miR-971	*pSS-DsRed*	F: CCGGGTACCGCAGCAGCCATCCACCAGCA<br>R: CCGTCTAGATCCTGTGGTGGTATCCCATTCCGA

Supplementary Table 1 (Continued)

miRNA	Vector	Primers (5' to 3')
miR-972	*pSS-DsRed*	F: CCGGGTACCAGTCTTGTCAATTCACCAGCATCGAA<br>R: CCGTCTAGATCCATTACTCGTAGAGCGAAAGGAGA
miR-973/974	*pSS-DsRed*	F: CCGGGTACCCCTTTCGCTCTACGAGTAATGGA<br>R: CCGTCTAGATGGTAAAACAGGAAGTTCTTTATCACG
miR-975/976/977	*pSS-DsRed*	F: CCGGGTACCTGTACTTGTCGGCACTGGAGTACTAA<br>R: CCGTCTAGATGGTAAAACAGGAAGTTCTTTATCACG
miR-978	*pSS-DsRed*	F: CCGGGTACCACCGCAAACCCCGTAGCAGG<br>R: CCGTCTAGAAGGGAAGGCACTCGCGCACT
miR-979	*pSS-DsRed*	F: CCGGGTACCTGAGGAACGATATCAGCAGTTGGGT<br>R: CCGTCTAGATGTGGACGTGGAAACGGAAACGG
miR-981	*pSS-DsRed*	F: CCGGGTACCCGTTTCTTGCCAAACCGGGTCCA<br>R: CCGTCTAGACCGACGATTCAAGGCAACCGGA
miR-982	*pSS-DsRed*	F: CCGGGTACCCATGTGTCGTCGCATCGAGAAGT<br>R: CCGTCTAGATGCAGCGTTATGCCATCCTTATCTCG
miR-983-1/983-2/984	*pSS-DsRed*	F: CCGGGTACCCGTCAACTCGGTGAGTGAATGGGA<br>R: CCGTCTAGAGAATGCGCCATTCAATGTCTATCTGA
miR-988	*pSS-DsRed*	F: GCGGCCGCTGGTATGAGGCTGACAAGCGTCG<br>R: GAGCTCGCAGCACATTGCACTCGCCC
miR-985	*pSS-DsRed*	F: CCGGGTACCTGGCCCAGTTTATTAGTGAATTGTGTG<br>R: CCGTCTAGAGCTTTTGGCTTTTATGTTTAC
miR-986	*pSS-DsRed*	F: CCGGGTACCTGGCCCTACCACTGCTGCTG<br>R: CCGTCTAGAACACAACACAAATCAAAGCCTCGTCA
miR-987	*pSS-DsRed*	F: CCGGGTACCGCAACCGCGTCGACAATCGC<br>R: CCGTCTAGATGTGTGGAAAATCAGTTTGAATGC
miR-989	*pSS-DsRed*	F: CCGGGTACCGACTTCAGGTGCAGCAA<br>R: CCGTCTAGAAGTCCGCAAAAACATATAAAATGC
miR-990	*pSS-DsRed*	F: CCGGGTACCCCCCCACTGACCGACTGACT<br>R: CCGTCTAGACCGGAATTACAGAGCCAAACCGCA
miR-991	*pSS-DsRed*	F: CCGGGTACCTCCCAAGTGCCTGGTATCAGCAAA<br>R: CCGTCTAGAGGTAAAAGACGGTTATGCCAATTTTGT
miR-993	*pSS-DsRed*	F: GGTACCTGGTTCTGTTCTGTGCGCGA<br>R: TCTAGAACGAGAACTGAACTCGTGCGTCT
miR-994	*pSS-DsRed*	F: CCGGGTACCCGCCAATTGCCGGGATCAGC<br>R: CCGTCTAGAAAACCGAAGGACCTTTTCATCCAGG
miR-995	*pSS-DsRed*	F: GCGGCCGCCGCGCCGAAAAGATTGGCGA<br>R: GAGCTCAGCAGGGAGATCTCTCGAATGGC
miR-996	*pSS-DsRed*	F: CCGGGTACCAAGCCCCAAACGGAAAACCCA<br>R: CCGTCTAGATAGTTGCTGCTGCTGCCGGG
miR-997	*pSS-DsRed*	F: CCGGGTACCTCCACCGAGCAGCTTGGACA<br>R: CCGTCTAGAACCGATTCAGCTCACGCGAAAGT
miR-998	*pSS-DsRed*	F: CCGGGTACCACCACCCAACCGACAACCCT<br>R: CCGTCTAGATTCTGCAGTTCGGGGCTGGG
miR-999	*pSS-DsRed*	F: CCGGGTACCCCCCGCCGATGCGACATCAT<br>R: CCGTCTAGAGTTGCGGTCCGGCCGTCTTC
miR-1000	*pSS-DsRed*	F: CCGGGTACCCGTACCTCGAAAGCGGGCCA<br>R: CCGTCTAGAATGGACGAGCTCCTGGGTGC
miR-1001	*pSS-DsRed*	F: CCGGGTACCTCAACCGAACACAAACCGAACTCA<br>R: CCGTCTAGAACCGTCCTCAGGGTCGGA
miR-1002	*pSS-DsRed*	F: GGTACCACAGGCGTATTCTTCAGCGTCAAT<br>R: TCTAGACAAGAGCATCCGCACGGGCT
miR-1003	*pSS-DsRed*	F: CCGGGTACCGAGTTCTGGCGGTGGCGGAC<br>R: CCGTCTAGATGCTGTGTTTCCGGCGGTGG
miR-1004	*pSS-DsRed*	F: CCGGGTACCGGACTCTCAGCCCGTTGGCG<br>R: CCGTCTAGATCACCTGCTTTCGTGCCACCAGCC
miR-1005	*pSS-DsRed*	F: CCGGGTACCTCGCACGTGATCGTTGGAGCC<br>R: CCGTCTAGAAGCACGAACATCGAGACCCGC
miR-1007	*pSS-DsRed*	F: CCGGGTACCACGGCGTGGGCATCACATCG<br>R: CCGTCTAGAAGCAGAAGGAGCTTGTTGCGCC
miR-1008	*pSS-DsRed*	F: CCGTCTACCTCGCGTCCCGCCAATTTCCC<br>R: CCGTCTAGAGTTTGCGATCCGCCGGCAAC
miR-1009	*pSS-DsRed*	F: CCGGGTACCATGCTGCTCTGCCGAGTCCT<br>R: CCGTCTAGAAGCTGCAGCGCAAAATGCGT
miR-1010	*pSS-DsRed*	F: CCGGGTACCGCTGCCGGAGCAGCGAATGA<br>R: CCGTCTAGAGGTGCGCCATTCATGGAAATCGT
miR-1011	*pSS-DsRed*	F: CCGGGTACCGCGCGTGGAGACGGCCATTA<br>R: CCGTCTAGATTCCGCCCGCTGATGTGCTG
miR-1012	*pSS-DsRed*	F: CCGGGTACCAGTTGCAGAGTGGGCGACACA<br>R: CCGTCTAGAGACCCGCGAACTCCGGATGG

Supplementary Table 1 (Continued)

miRNA	Vector	Primers (5' to 3')
miR-1013	*pSS-DsRed*	F: CCGGGTACCGCACGGGATTCCGATGGACGC<br>R: CCGTCTAGATTGGCCAGAAAGGGCGGCTG
miR-1015	*pSS-DsRed*	F: CCGGGTACCCCGGCGAGCACTGCGACTAC<br>R: CCGTCTAGAGGGAATTGCTTTTGGTGACTTACCGGA
miR-1016	*pSS-DsRed*	F: CCGGGTACCCGAGGAGCTCGCCGATCCTG<br>R: CCGTCTAGACATCTCGACCAGACTCGCGCA
miR-1017	*pSS-DsRed*	F: CCGGGTACCACGGCTCCATCAACGGGGCT<br>R: CCGTCTAGACTCGCCAGAATCGGCGGGC
miR-2279	*pSS-DsRed*	F: CCGGGTACCTGCAGTTGGAGTCGATGATAAAGTCG<br>R: CCGTCTAGATCGGTTGACGCCTTCGGACT
miR-2280	*pSS-DsRed*	F: CCGGGTACCTGCCTGCAGTTCCATGGGCG<br>R: CCGTCTAGACCAAGAGCTCGGTCAAACGGCA
miR-2282	*pSS-DsRed*	F: CCGGGTACCCTCAAGACGGGCCCACGCAG<br>R: CCGTCTAGACGTGTCACGAACACACCCCCA
miR-2283	*pSS-DsRed*	F: CCGGGTACCAGTTTGCGACGCCCATTGGTGA<br>R: CCGTCTAGAAGCATTGGCAAGCCATTTACCGA
miR-2281	*pSS-mCherry*	F: GCGGCCGCGAATGGCGCTGGAGGAGCGT<br>R: GAGCTCTGCCACATCGGGCAGCTGAA
miR-2491	*pSS-mCherry*	F: GGTACCATGAACAGAAGTGTGAACGTGATTGGT<br>R: TCTAGAATGGTGAGTGCCACCCGCAC
miR-2492	*pSS-mCherry*	F: GGTACCAGGGGTTAAAGGGGCGGGCT<br>R: TCTAGAGGTCAAACCCGATGACGATGATGA
miR-2494	*pSS-mCherry*	F: GGTACCGGTGCATGACATCGCAGAGCG<br>R: TCTAGAGGGGGCGTCGTGACCTTTCC
miR-2495	*pSS-mCherry*	F: GGTACCTGTTGCATCGTGAGGCATCTTAGCTC<br>R: TCTAGAGCTCGCCGCACTCCACTACG
miR-2496	*pSS-mCherry*	F: GGTACCGGTTGAAATGCGCTAATGGGGCG<br>R: TCTAGAGCACACACACAGCATACACACACA
miR-2497	*pSS-mCherry*	F: GGTACCTGCTGGAGGACCAGAGTCCTGT<br>R: TCTAGACCTCTCCGCTCAGCATTGGCA
miR-2499	*pSS-mCherry*	F: GGTACCGTGCTGCCACTTGCTGGCGA<br>R: TCTAGAAGCGAACACAGCCGCACACG
miR-2500	*pSS-mCherry*	F: GGTACCTGGGCAGGAGGCAGGCCAAT<br>R: TCTAGACGGCGAAGGAGAGTAGGTCGC
miR-2501	*pSS-mCherry*	F: GGTACCACGATGCGGTGCCCAGACAG<br>R: TCTAGAAGGCAAACCATTGAACGTCCGCT
miR-1	*pUAST-DsRed*	
miR-5	*pUAST-DsRed*	
miR-9a	*pUAST-DsRed*	
miR-10	*pUAST-DsRed*	
miR-12/283/304	*pUAST-DsRed*	
miR-33	*pUAST-DsRed*	
miR-79	*pUAST-DsRed*	
miR-87	*pUAST-DsRed*	
miR-92a	*pUAST-DsRed*	
miR-92b	*pUAST-DsRed*	
miR-124	*pUAST-DsRed*	
miR-219	*pUAST-DsRed*	
miR-252	*pUAST-DsRed*	
miR-263a	*pUAST-DsRed*	
miR-263b	*pUAST-DsRed*	
miR-274	*pUAST-DsRed*	

Supplementary Table 1 (Continued)

miRNA	Vector	Primers (5' to 3')
miR-276a	*pUAST-DsRed*	
miR-276b	*pUAST-DsRed*	
miR-277	*pUAST-DsRed*	
miR-278	*pUAST-DsRed*	
miR-279	*pUAST-DsRed*	
miR-280	*pUAST-DsRed*	
miR-282	*pUAST-DsRed*	
miR-283	*pUAST-DsRed*	
miR-284	*pUAST-DsRed*	
miR-285	*pUAST-DsRed*	
miR-286	*pUAST-DsRed*	
miR-287	*pUAST-DsRed*	
miR-288	*pUAST-DsRed*	
miR-303	*pUAST-DsRed*	
miR-304	*pUAST-DsRed*	
miR-305	*pUAST-DsRed*	
miR-307a	*pUAST-DsRed*	
miR-308	*pUAST-DsRed*	
miR-309	*pUAST-DsRed*	
mIR-310	*pUAST-DsRed*	
miR-310/311/312/313	*pUAST-DsRed*	
miR-311	*pUAST-DsRed*	
miR-312	*pUAST-DsRed*	
miR-313	*pUAST-DsRed*	
miR-314	*pUAST-DsRed*	
miR-315	*pUAST-DsRed*	
miR-317	*pUAST-DsRed*	
miR-318	*pUAST-DsRed*	
miR-iab-4	*pUAST-DsRed*	

Note: All *pUAST-DsRed* vectors were obtained from the Drosophila RNAi Screening Center and modified by inserting a minimal attB site.

Supplementary Table 2. The changes in wing length relative to the *Dilp2-Gal4* control induced by over-expression of miRNAs

miRNA	Average wing length (mm)	s.e.m.	*P*-value	miRNA	Average wing length (mm)	s.e.m.	*P*-value
miR-972	1.449363636	0.00441	1.09E-11	*miR-973/974*	1.412151515	0.003631	7.18E-05
miR-289	1.4473	0.004022	4.32E-13	*miR-284*	1.422769231	0.009314	0.000622
miR-278	1.443590909	0.006825	9.76E-09	*miR-288*	1.420454545	0.007462	0.000221
miR-312	1.438826087	0.005028	3.43E-09	*miR-1015*	1.41625	0.006674	0.0022
miR-2494	1.438	0.006379	3.20E-06	*miR-2282*	1.416055556	0.007318	0.001122
miR-994	1.43425	0.006152	1.50E-07	*miR-317*	1.415842105	0.005779	0.000453
miR-307a	1.432142857	0.004126	4.96E-08	*miR-277*	1.41555	0.0075	0.001304
miR-309	1.431785714	0.008067	1.62E-05	*miR-2499*	1.415083333	0.005683	0.002465
miR-970	1.431115385	0.005968	3.80E-07	*miR-303*	1.4144	0.006903	0.002247
miR-990	1.4304	0.00499	5.99E-08	*miR-2279*	1.414	0.006982	0.004481
miR-iab-4	1.429777778	0.006716	0.000149	*miR-311*	1.4135	0.006679	0.001607
miR-985	1.42952	0.005193	2.61E-07	*miR-12/283/304*	1.413133333	0.005857	0.002337
miR-287	1.429263158	0.005891	2.30E-06	*miR-997*	1.413	0.004325	0.001982
miR-281-2	1.4283	0.00745	1.33E-05	*miR-310/311/312/313*	1.411266667	0.008163	0.008481
miR-929	1.427166667	0.005064	4.28E-05	*miR-1007*	1.410952381	0.005991	0.002466
miR-286	1.426035714	0.005932	3.28E-06	*miR-14*	1.41076	0.00457	0.000763
miR-982	1.423727273	0.005026	5.56E-06	*miR-124*	1.4103	0.004953	0.002001
miR-285	1.423333333	0.007436	9.92E-05	*miR-280*	1.4102	0.004189	0.001437
miR-310	1.423166667	0.005835	3.32E-05	*miR-308*	1.410105263	0.004756	0.002249
miR-274	1.423058824	0.006489	6.67E-05	*miR-979*	1.41	0.004872	0.001904
miR-986	1.42162963	0.005788	2.01E-05	*miR-969*	1.409869565	0.00507	0.001839
miR-955	1.421	0.005799	4.00E-05	*miR-995*	1.409608696	0.006856	0.005717
miR-1016	1.420409091	0.00641	8.98E-05	*miR-314*	1.409586207	0.003496	0.000348
miR-92a	1.41952	0.005601	4.58E-05	*miR-1005*	1.409391304	0.006529	0.005134
miR-281-1	1.419	0.005378	8.57E-05	*miR-305*	1.409153846	0.005155	0.002115

Red : wing length is longer than the control. s.e.m. : standard error of the mean.

SupplementaryTable 2 (Continued)

miRNA	Average wing length (mm)	s.e.m.	*P*-value	miRNA	Average wing length (mm)	s.e.m.	*P*-value
miR-2497	1.409	0.005544	0.004211	*miR-987*	1.396923077	0.006798	0.16351
miR-34	1.408913043	0.00667	0.006465	*miR-283*	1.39613205	0.007954	0.306471
miR-998	1.408851852	0.005415	0.002756	*miR-1012*	1.393916667	0.008518	0.375946
miR-959/960/961/962	1.408125	0.004637	0.002559	*miR-92b*	1.393454545	0.009856	0.427951
miR-2496	1.408090909	0.008118	0.02884	*miR-31a*	1.392642857	0.006777	0.402058
miR-981	1.407774194	0.004803	0.002327	*miR-2491*	1.392363636	0.003163	0.422538
miR-978	1.407083333	0.007405	0.015906	*miR-2283*	1.392347826	0.00599	0.358257
miR-1017	1.405647059	0.007413	0.026916	*miR-219*	1.391555556	0.006948	0.53416
miR-79	1.40516	0.004914	0.008523	*miR-276a*	1.389448276	0.004282	0.508868
miR-2492	1.404133333	0.006964	0.040317	*miR-184*	1.389185185	0.006589	0.608004
miR-1011	1.404	0.005807	0.020283	*miR-975/976/977*	1.38764	0.004961	0.709021
miR-999	1.40332	0.003598	0.008922	*miR-8*	1.387619048	0.005442	0.727286
miR-313	1.4032	0.010212	0.105925	*miR-1010*	1.3867	0.009588	0.8558
miR-2280	1.403157895	0.004213	0.020378	*Dilp2-Gal4*	1.38483871	0.005379	1
miR-282	1.402772727	0.006091	0.033189	*miR-1003*	1.384692308	0.006912	0.987714
miR-33	1.400954545	0.006077	0.054397	*miR-304*	1.384	0.009831	0.941239
miR-315	1.40075	0.013339	0.211629	*miR-1008*	1.38345	0.006482	0.870644
miR-210	1.3996	0.007185	0.116229	*miR-100*	1.383153846	0.007926	0.863882
miR-10	1.399466667	0.005378	0.096416	*miR-5*	1.3824	0.003484	0.803683
miR-2495	1.398789474	0.008491	0.15055	*miR-1013*	1.381571429	0.006438	0.699419
miR-137	1.3984	0.004109	0.108342	*miR-2501*	1.38075	0.007768	0.720603
miR-996	1.398235294	0.004708	0.103924	*miR-1001*	1.380357143	0.007436	0.637423
miR-87	1.398166667	0.005806	0.116684	*miR-133*	1.380076923	0.010283	0.65624

Yellow box : wing length of the *Dilp2-Gal4* control. s.e.m. : standard error of the mean.

Supplementary Table 2 (Continued)

miRNA	Average wing length (mm)	s.e.m.	*P*-value	miRNA	Average wing length (mm)	s.e.m.	*P*-value
miR-318	1.379933333	0.006159	0.582381	*miR-932*	1.34675	0.007248	0.001712
miR-2a-1	1.37985	0.006806	0.566493	*miR-375*	1.341	0.008968	0.000532
miR-13b-2	1.379444444	0.007469	0.620357	*miR-4*	1.338	0.014555	0.000807
miR-1009	1.378764706	0.005192	0.464421	*miR-1*	1.347214286	0.00481	3.12E-06
miR-1006	1.377916667	0.0063	0.47127	*miR-7*	1.344388889	0.007092	3.78E-05
miR-125	1.377833333	0.005533	0.374182	*miR-11*	1.340928571	0.006935	2.40E-05
miR-989	1.377392857	0.003239	0.252819	*miR-1002*	1.337555556	0.00603	6.63E-05
miR-983-1/-2/984	1.374103448	0.006948	0.223331	*let-7*	1.330769231	0.002199	1.14E-07
miR-971	1.372666667	0.006528	0.259518	*miR-3/309*	1.3294	0.005079	6.13E-08
miR-1004	1.371777778	0.009929	0.256049	*miR-9a*	1.328263158	0.004158	1.64E-09
miR-6-1/6-2/6-3	1.371	0.010427	0.250019	*miR-9b/79/306*	1.290388889	0.005674	3.98E-15
miR-276b	1.36975	0.005698	0.115279				
miR-263a	1.368956522	0.004962	0.040817				
miR-991	1.368608696	0.003739	0.025062				
miR-2500	1.367636364	0.004565	0.077141				
bantam	1.365909091	0.008292	0.073576				
miR-2a-2	1.365333333	0.005169	0.019954				
miR-1000	1.362074074	0.003907	0.001511				
miR-2281	1.361846154	0.006645	0.018392				
miR-988	1.361461538	0.010214	0.03287				
miR-252	1.36	0.003535	0.001319				
miR-263b	1.359466667	0.004775	0.000839				
miR-2b-2	1.356636364	0.007238	0.007592				
miR-279	1.3553	0.005777	0.000689				
miR-993	1.351666667	0.009049	0.004978				

Blue : wing length is shorter than control. s.e.m. : standard error of the mean.

Supplementary Table 3. Synthetic *miRNA-9a* sequences used in this study

miRNA duplex	Sequence (5' to 3')
miR-9a scrambled	sense: CCGAACACAAAACAUUAUGGAA<br>antisense: UUCCAUAAUGUUUUGUGUUCGGG
Biotinylated-miR-9a	sense: Biotin - UCUUUGGUUAUCUAGCUGUAUGA – Ph<br>antisense: ACGUUAUACAGCUAGAUAACCAAA - Ph
miR-9a mimic	sense: UCUUUGGUUAUCUAGCUGUAUGA

Biotin : biotinylation. Ph : phosphorylation

Supplementary Table 4. Other PCR primers used in this study

PCR primer	Product size	Primer sequence (5' to 3')
RP49	122bp	F: AGGGTATCGACAACAGAGTG<br>R: CACCAGGAACTTCTTGAATC
Dilp2	256bp	F: GTATGGTGTGCGAGGAGTAT<br>R: TGAGTACACCCCCAAGATAG
Dilp3	288bp	F: AAGCTCTGTGTGTATGGCTT<br>R: AGCACAATATCTCAGCACCT
Dilp5	282bp	F: AGTTCTCCTGTTCCTGATCC<br>R: CAGTGAGTTCATGTGGTGAG
d4E-BP	457bp	F: GATCACCAGGAAGGTTGTC<br>R: GGTCAATATGACCGAGAGAA
tubulin	182bp	F: ACTGCAGCATCCTGTGAACC<br>R: TGGGAACATTTCCGTTTGAT
sNPFR1	164bp	F: GGGCCATTTCGCATATTTAC<br>R: ATTTAATTCCGTGCGACTGG
senseless	128bp	F: TGGCAGCTAAACGTACCAAA<br>R: GATCGTATAAATAAATGTGG
GAPDH	154bp	F: ATGGAGCCTCCTTCACCTG<br>R: TCTCCCTCACAATTCCATCC
Actin	178bp	F: ATGTGGCAGAAGCAGGAAAG<br>R: CGTATGTGGGAGTGTGGACT
NPY2R	150bp	F: GGGGGATGTTAGCAATTTTC<br>R: TCAGCATCCAGAGAAGTGCA
Foxg1	327bp	F: CCAGATTTCCATGTGTGCAG<br>R: GCCAAACTAAACAACCTTCC
Dilp2-Gal80^{ts}	859bp	F: CGGGGTACCCCAACACACACATTC<br>R: CTAGCTAGCTGGTTATGGGTTTACTG

Supplementary Table 5. CLIP assay results showing total number of clones analyzed in *Drosophila* S2 cells and rat INS-1 cells.

Cell Type	Scramble			miR-9a duplex		
**S2 cell**	**# of clones sequenced**	**Deletion**	**Deletion (%)**	**# of clones sequenced**	**Deletion**	**Deletion (%)**
Trial1	10	0	0	10	0	0
Trial2	6	0	0	6	1	17
Trial3	20	0	0	20	3	15
Trial4	9	0	0	10	0	0
Trial5	2	0	0	10	1	10
Total	47	0	0	56	5	8
**INS-1 cell**	**# of clones sequenced**	**Deletion**	**Deletion (%)**	**# of clones sequenced**	**Deletion**	**Deletion (%)**
Trial1	12	1	8	12	3	25
Trial2	10	2	20	10	4	40
Trial3	10	1	10	10	2	20
Trial4	20	0	0	20	3	15
Total	52	4	10	52	12	25
